# A new non-classical fold of varroa odorant-binding proteins reveals a wide open internal cavity

**DOI:** 10.1038/s41598-021-92604-2

**Published:** 2021-06-23

**Authors:** Beatrice Amigues, Jiao Zhu, Anais Gaubert, Simona Arena, Giovanni Renzone, Philippe Leone, Isabella Maria Fischer, Harald Paulsen, Wolfgang Knoll, Andrea Scaloni, Alain Roussel, Christian Cambillau, Paolo Pelosi

**Affiliations:** 1 Architecture et Fonction des Macromolécules Biologiques (AFMB, UMR 6098), Centre National de la Recherche Scientifique (CNRS), Aix-Marseille Université (AMU), Campus de Luminy, Case 932, 13288 Marseille Cedex 09, France; 2 Biosensor Technologies, Austrian Institute of Technology GmbH, Konrad-Lorenz Straße, 24, 3430 Tulln, Austria; 3 Proteomics and Mass Spectrometry Laboratory, ISPAAM, National Research Council, 80147 Naples, Italy; 4 Faculty of Biology, Institute of Molecular Physiology, Johannes Gutenberg-Universität Mainz, 55099 Mainz, Germany; 5 Department of Physics and Chemistry of Materials, Faculty of Medicine/Dental Medicine, Danube Private University, Krems, Austria

**Keywords:** Biochemistry, Structural biology

## Abstract

Odorant-binding proteins (OBPs), as they occur in insects, form a distinct class of proteins that apparently has no closely related representatives in other animals. However, ticks, mites, spiders and millipedes contain genes encoding proteins with sequence similarity to insect OBPs. In this work, we have explored the structure and function of such non-insect OBPs in the mite *Varroa destructor*, a major pest of honey bee. Varroa OBPs present six cysteines paired into three disulphide bridges, but with positions in the sequence and connections different from those of their insect counterparts. VdesOBP1 structure was determined in two closely related crystal forms and appears to be a monomer. Its structure assembles five α-helices linked by three disulphide bridges, one of them exhibiting a different connection as compared to their insect counterparts. Comparison with classical OBPs reveals that the second of the six α-helices is lacking in VdesOBP1. Ligand-binding experiments revealed molecules able to bind only specific OBPs with a moderate affinity, suggesting that either optimal ligands have still to be identified, or post-translational modifications present in the native proteins may be essential for modulating binding activity, or else these OBPs might represent a failed attempt in evolution and are not used by the mites.

## Introduction

Odorant-binding proteins (OBPs) are carriers of pheromones and odorants in vertebrates and insects^1–4^. Insect OBPs, which represent a class of proteins structurally different^5, 6^ from those of vertebrates^7, 8^, have been detected so far only in Hexapoda^4, 9^. They are polypeptides of 120–140 amino acids, folded in a compact structure generally made of six α-helical domains. A pattern of six conserved cysteines paired in three interlocked disulphide bridges is a key feature of these proteins^10–12^. A single hydrophobic pocket binds small organic volatile compounds, such as pheromones and general odorants, with dissociation constants of the micromolar order^13^. Besides OBPs, other soluble carriers such as chemosensory proteins (CSPs), Nieman-Pick C2 (NPC2) proteins and lipocalins, may be involved in chemical communication of arthropods^4, 9, 14–16^. In particular, NPC2 proteins are expressed with several members in all arthropods and represented until a couple of years ago the only known class of putative semiochemical carriers in Chelicerata and Crustacea.

However, recent work unveiled the presence of proteins with some sequence similarity to insect OBPs in spiders, ticks and mites, as well as in Myriapoda, which have been tentatively named “OBP-like”^17–21^. Such polypeptides exhibit values of amino acid identity with insect OBPs around 10–15%. However, such low similarity can be significant when compared with the low values of conserved amino acids between the OBPs of different insect Orders. Given their phylogenetic relationship with their insect counterparts, we shall simply refer to these proteins as OBPs. Members of this family have not been found so far in Crustacea nor in other species of living organisms.

**Figure 1:**
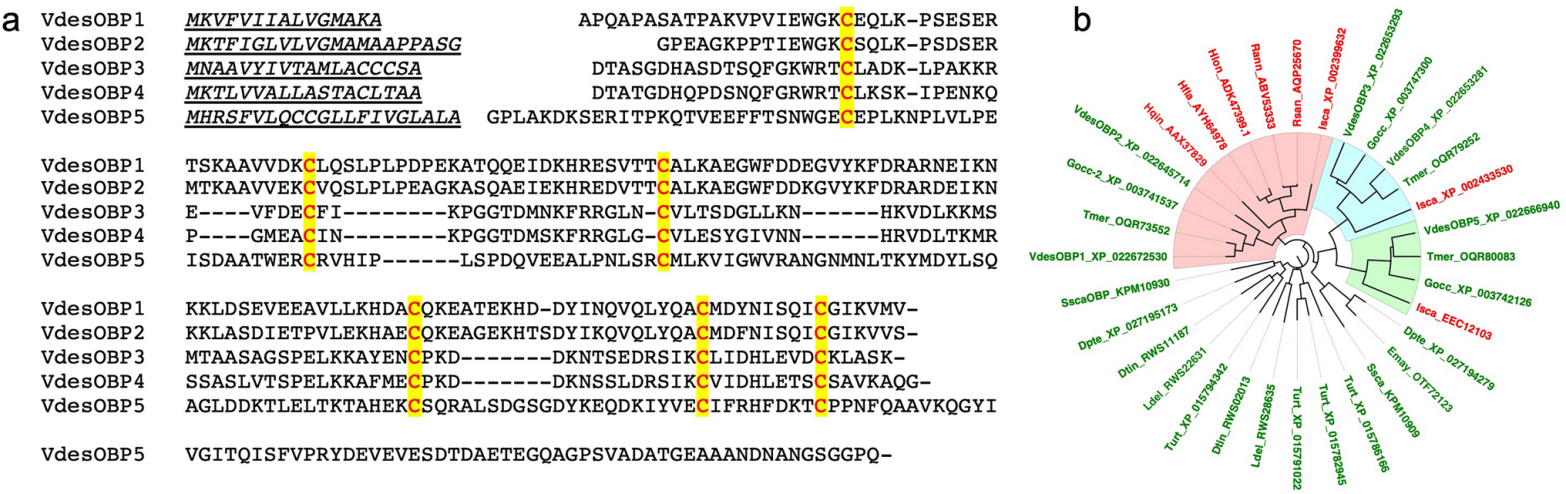
(**a**) Alignment of the five OBPs of *V. destructor*. The six conserved cysteines are highlighted. VdesOBP1 and VdesOBP2 are very similar (72% identity), as VdesOBP3 and VdesOBP4 (60%), but proteins of the two groups share only about 15% of their residues between them as well as with VdesOBP5. The six conserved cysteines are highlighted and in red font. Signal peptides are underlined. (**b**) Phylogenetic tree of tick and mite OBPs. Vdes: *Varroa destructor*; Turt: *Tetranychus urticae*; Hqin: *Haemaphysalis qinghaiensis*; Hfla: *Haemaphysalis flava*; Hlon: *Haemaphysalis longicornis*; Isca: *Ixodes scapularis*; Gocc: *Galendromus occidentalis*; Tmer: *Tropilaelaps mercedesae*; Rann: *Rhipicephalus annulatus*; Rsan: *Rhipicephalus sanguineus*; Ldel: *Leptotrombidium delicense*; Dtin: *Dinothrombium tinctorium*; Dpte: *Dermatophagoides pteronyssinus*; Ssca: *Sarcoptes scabiei*; Emay: *Euroglyphus maynei*. All sequences used to build the tree are reported in Supplementary Table S1 online. Names of ticks are in red, those of mites are in green. Highlighted clades contain VdesOBP1 and VdesOBP2 (pink), VdesOBP3 and VdesOBP4 (light blue), VdesOBP5 (light green).

In order to understand the role of OBPs in Chelicerata, we have focused our attention on the mite *Varroa destructor*, the well-known honey bee parasite. The genome of this species contains 5 genes encoding members of the OBP family^18, 19, 22^. The sequences of the five encoded proteins are aligned in Fig. 1a. At the mature protein level, VdesOBP1 and VdesOBP2 are very similar to each other with 72% identity. Also VdesOBP3 and VdesOBP4 are closely related sharing 60% of their residues, but the identity levels drop to 15% or less between the two groups and with VdesOBP5. Orthologues of these sequences are found also in other mites. Some representative examples are reported in the phylogenetic tree of Fig. 1b, where the three clusters (OBP1-2, OBP3-4 and OBP5) are clearly separated. A number of sequences from other ticks and mites fall into different groups. In particular, all four OBPs of the spider mite *Tetranychus urticae* belong to a cluster well distinct from the three highlighted for varroa. Besides, the four OBPs of *T. urticae* are much more similar to each other with identities around 40%. This suggests that different mites may have adopted different sub-classes of OBPs for their chemoreception systems, although we cannot exclude that such differences might be partly due to incomplete annotation. In any case, OBPs are present in each species of Chelicerata with a small number of genes, poorly conserved between species and highly divergent within the same species. Unfortunately, due to only few genomes available and their poor annotation, the picture provided by this tree is to be considered as partial and preliminary. The amino acid sequences used to build the phylogenetic tree are reported in Supplementary Table S1 online.

Transcriptomic studies reported VdesOBP2 (XP_022645714) as the member with the highest expression in the forelegs^18^, while the gene encoding VdesOBP3 (XP_022653293) was found to be upregulated in the forelegs, where chemosensory appendages are located, with respect to the second pair of legs^19^. At the proteomic level, four OBPs were detected: VdesOBP1 (XP_022672530), VdesOBP2, VdesOBP3 and VdesOBP4 (XP_022653281), the last two being more highly expressed in the forelegs than in hindlegs^22^.

In this work, we have expressed and characterised the OBPs of this mite. We have also solved the crystal structure of VdesOBP1, which has a fold different from that of insect OBPs.

## Results

### Protein expression and purification

First we confirmed the five OBP sequences reported in the NCBI database by cloning the five genes from RNA extracted from whole individuals. Sequences were identical to the published ones at the amino acid level, while few single base substitutions were likely due to differences between the population of mites used in our study with respect to those used for the sequences stored in the NCBI database.

**Figure 2:**
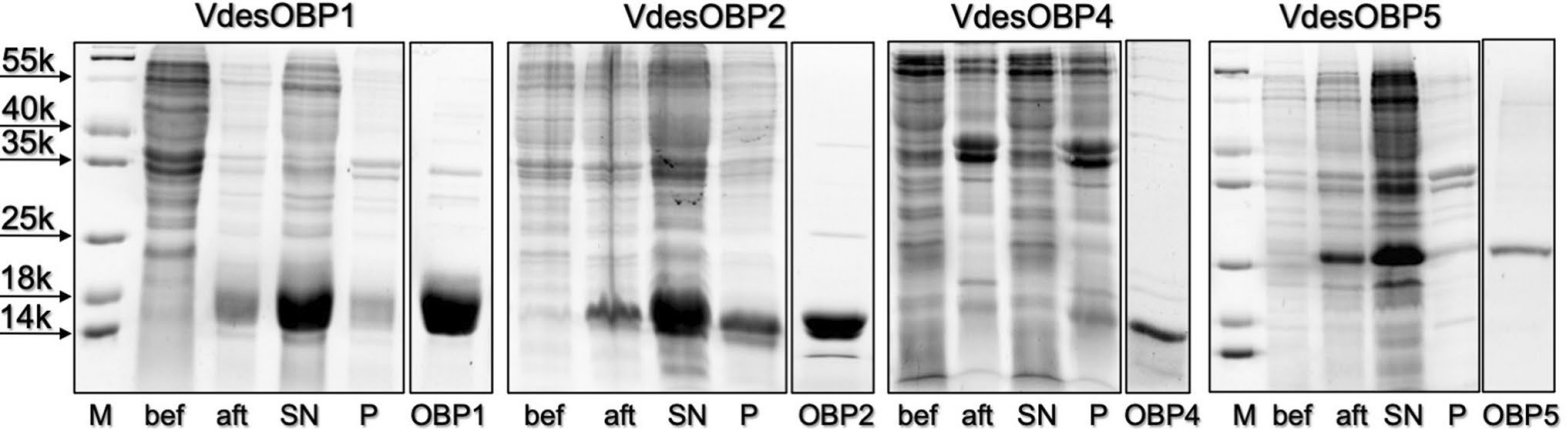
Expression and purification of four OBPs of V. *destructor*. While VdesOBP1, VdesOBP2 and VdesOBP5 were easily produced in bacteria, VdesOBP3 and VdesOBP4, which are structurally related, proved difficult to express. In fact, VdesOBP4 was obtained only in very low yield, while we could not observe expression of VdesOBP3 in any of the different constructs adopted. M: Molecular weight markers; bef: crude sample before culture induction; aft: crude sample after induction with IPTG; SN: supernatant after lysis and centrifugation; P: pellet after lysis and centrifugation; OBP: sample of purified protein.

The mature proteins, containing an additional methionine present at the N-terminus, were expressed in bacteria, following standard protocols, as reported in the Methods section. We have successfully obtained VdesOBP1, VdesOBP2, VdesOBP4 and VdesOBP5 in medium to good yields, but we were unable to express VdesOBP3 in amounts adequate for purification and consequently for its characterization. The proteins were purified by two or three chromatographic steps on anion-exchange columns, such as DE-52 and HiPrep-Q. Figure 2 reports a summary of expression and purification for the four OBPs.

### Cysteine pairing assessment

**Figure 3:**
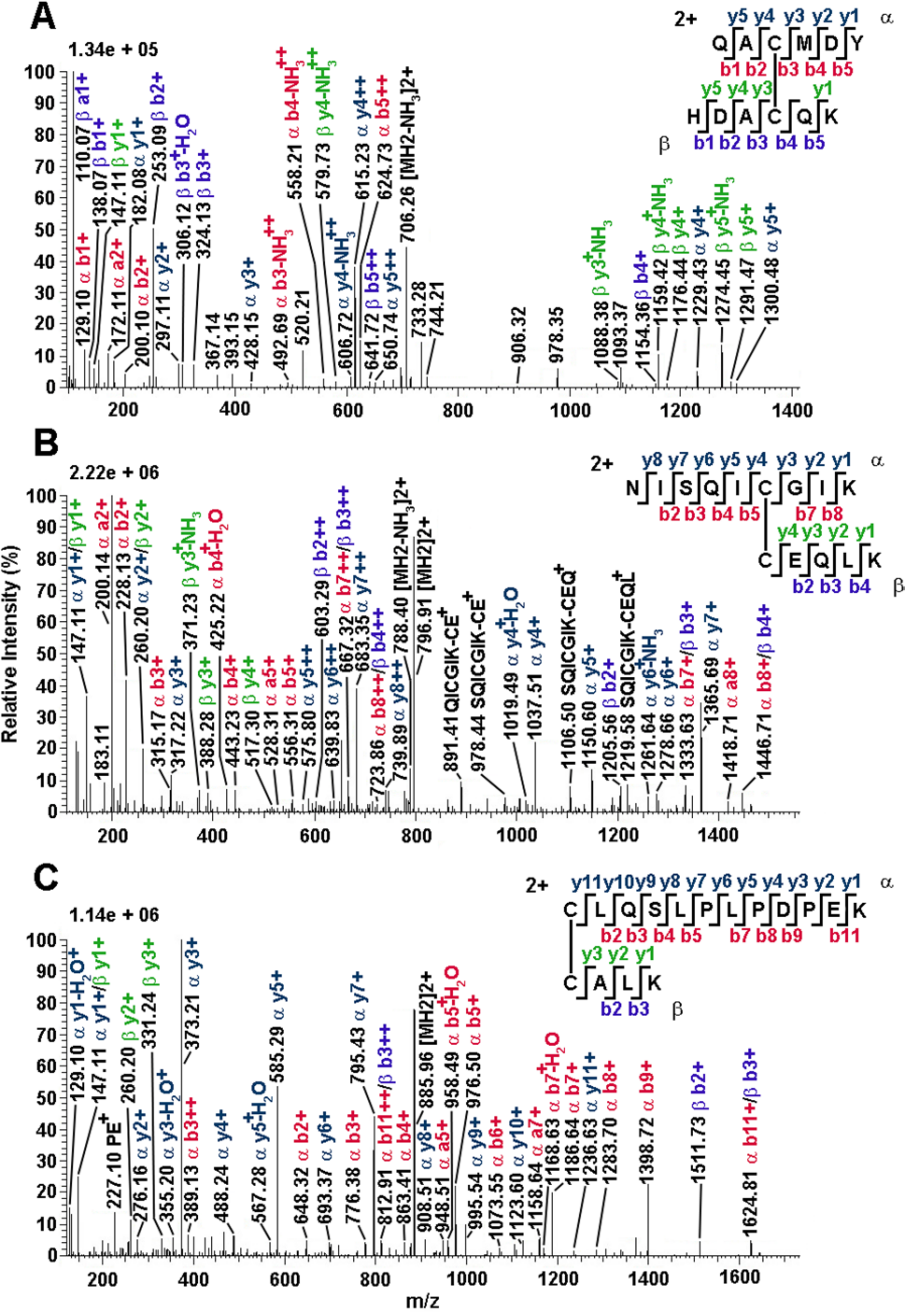
Fragmentation spectra of disulfide-bridged peptides identified in the tryptic-chymotryptic digest of *V. destructor* VdesOBP1 as revealed by nanoLC-ESI-Q-Orbitrap-MS/MS analysis. The fragments are highlighted in different colours depending on peptides present in S–S-linked species, and on the corresponding b and y ion series. Complete data on disulphide-bridged peptides are reported in Supplementary Table S2 online.

VdesOBP1-5 contains six cysteines occurring at conserved positions (Fig. 1), although in a pattern different from the one typical of insect OBPs. This fact suggested possible differences in the cysteine pairing with respect to insects^10–12^. To assess this issue, VdesOBP1, VdesOBP2 and VdesOBP5 were subjected to extensive alkylation with iodoacetamide under non-reducing denaturing conditions; after purification, they were then digested with trypsin followed by a second treatment with chymotrypsin. Resulting digests were analyzed by nanoLC-ESI-Q-Orbitrap-MS/MS, and S–S-crosslinked peptides were assigned using dedicated bioinformatics procedures (Supplementary Table S2 online). Examples of mass spectra of disulphide-bridged peptides from VdesOBP1 and VdesOBP5 digests are reported in Fig. 3 and Supplementary Figure S1 online, respectively.

These results and the absence of carboxyamidomethylated peptides in the protein digests demonstrated that all cysteines present in VdesOBPs are involved in disulphide bridges. Surprisingly, the ascertained, conserved disulphide pattern of VdesOBPs was unlike that of insect OBPs. In fact, a C1–C6, C2–C3 and C4–C5 arrangement was identified in VdesOBPs, which was completely different form the C1–C3, C2–C5, C4–C6 typical of insect OBPs^10–12^. This phenomenon brings two major consequences in the structure of VdesOBPs: (1) the pairing of adjacent cysteines should make *V. destructor* proteins more flexible and less compact than insect OBPs; (2) linking C1–C6 brings together the protein N- and C-termini, while these two ends point generally in opposite directions in insect OBPs.

### Protein characterization in solution

The protein oligomeric state of VdesOBP1 was characterized in solution at different pH values, namely 4.5, 7.0 and 9.7, using HPLC with multi-angle laser light scattering /UV/ refractometry (SEC–MALLS-RI) coupled detectors (Wyatt, Santa-Barbara, USA). The results point to a monomeric state, with measured mass values of 15,708, 16,143 and 15,306 Da (16.496 kDa theoretical mass), respectively (Supplementary Fig. S2 online).

### Overall structure of VdesOBP1

The three-dimensional structure of VdesOBP1 was solved by sulphur single-wavelength anomalous dispersion (SSAD) with a wavelength λ = 1.512 Å on beamline proxima 2A at SOLEIL synchrotron (Saint-Aubin, France). Data were collected at 1.968 Å resolution on a P3_2_21 crystal form and a second dataset was collected at 1.81 Å resolution with wavelength λ = 0.98011 Å (Table 1). Another crystal form was collected, with space group P2_1_ at 1.2 Å resolution, and its structure was determined by molecular replacement using the P3_2_21 crystal form. VdesOBP1 crystal forms P3_2_21 and P2_1_ were crystallized at pH values of 7.5 and 9.5, respectively. They contain one and two molecules in the asymmetric unit, respectively. After refinement, the final R/Rfree values were 18.6/22.6% and 18.0/20.1% for the two crystal forms, respectively (Table 1).

**Table 1:** Data collection and refinement statistics.

**Data collection**		
Beamline	SOLEIL Proxima2	SOLEIL Proxima1
PDB ID	7NYJ	7NZA
Space group	P3_2_21	P2_1_
Cell parameters, a, b, c (Å), γ/β (°)	53.8, 53.8, 91.7 γ = 120°	31.6, 113.0, 41.8 β = 98.95°
Wavelength (Å)	0.98011	0.97856
Resolution (Å)	46.6–1.81 (1.95–1.81)	40.09–1.20 (1.27–1.20)
Rmerge^a^ (%)	7.95 (110)	6.5 (88.5)
Mean((I)/sd(I))^a^	13.26 (1.87)	13.15 (1.19)
cc(1/2)	99.9 (76.3)	99.8 (78.8)
No. of observations^a^	284,352 (57,758)	634,776 (86,086)
No. of unique reflections^a^	13,957 (2889)	87,891 (13,358)
Completeness^a^ (%)	100.0 (100.0)	97.2 (91.4)
Multiplicity^a^	20.4 (20.0)	7.0 (6.4)
**Refinement**		
Resolution (Å)	46.59–1.81 (1.857–1.810)	38.8–1.20 (1.23–1.20)
No. of reflections^a^	13,827 (1009)	83,470 (5555)
atoms:protein/ligands/solvent	2289/36/393	2325/36/340
No. of test set reflections	728 (53)	4402 (308)
Rwork/Rfree^a^ (%)	18.6 (28.6)/22.6 (31.0)	18.0 (33.0)/20.1(34.1)
rmsd bonds (Å)/angles (°)	0.015/1.83	0.018/2.20
B-factors (Å^2^)	40.5	19.0
Ramachandran: favored/allowed (%)	98.5/1.5	96.0/4.0

^a^Refers to the highest-resolution bin.

**Figure 4:**
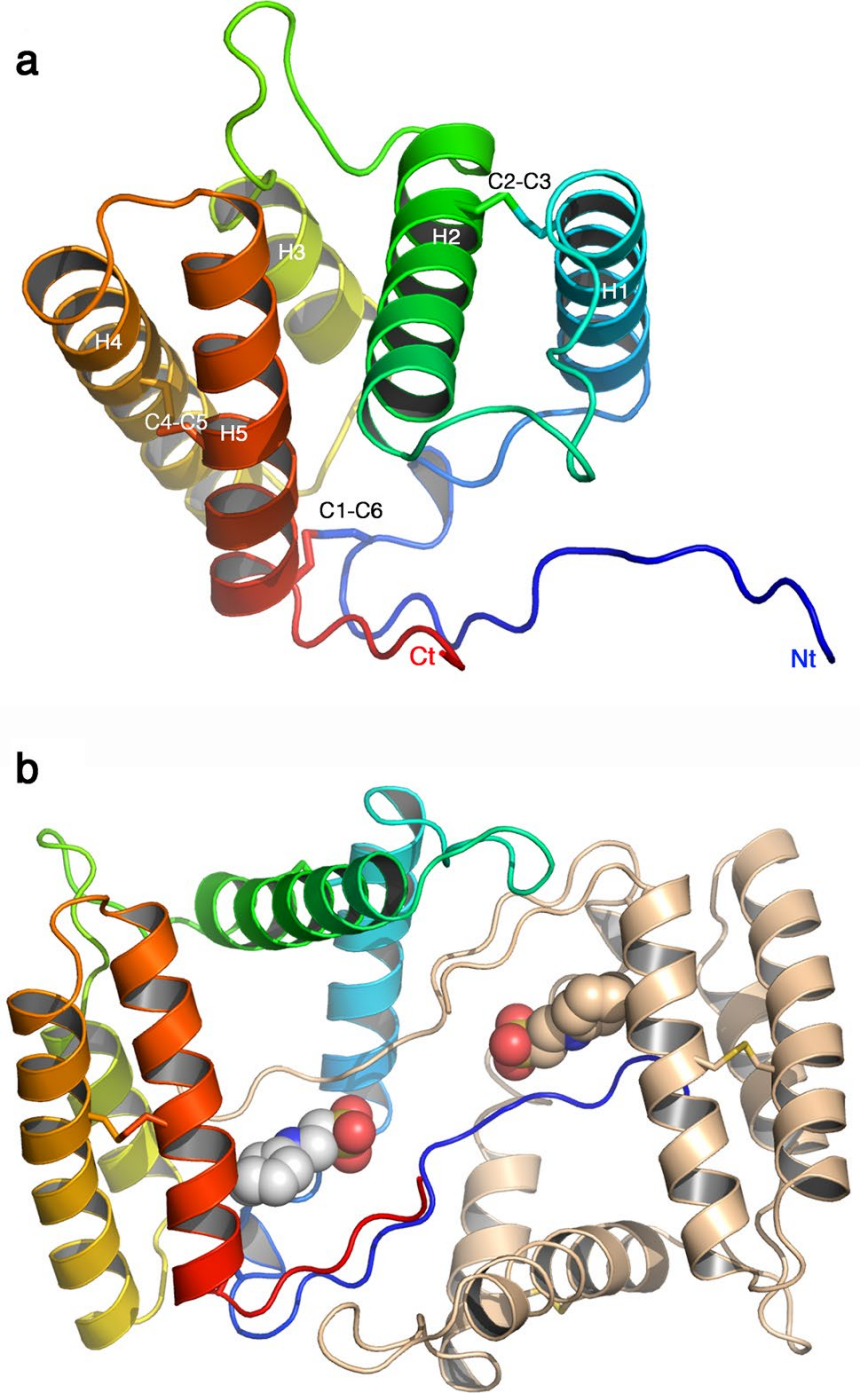
Crystal structure of VdesOBP1 form P2_1_. (**a**) Ribbon view of a monomer displaying helices 1–5 and disulphide bridges. The polypeptide chain is rainbow coloured, from blue (N-terminus) to red (C-terminus). The disulphide bonds are identified by the secondary structures they belong to. (**b**) Ribbon view of the crystallographic dimer. One monomer is rainbow coloured, the other one is coloured brown. The serendipitously bound buffer molecule 2-(N-cyclohexylamino)-ethane sulfonic acid (NCES) is displayed as atomic spheres (C: white, N: blue and O: red). Figure made with PyMOL (version 1.5.0.2. http://www.pymol.org).

**Figure 5:**
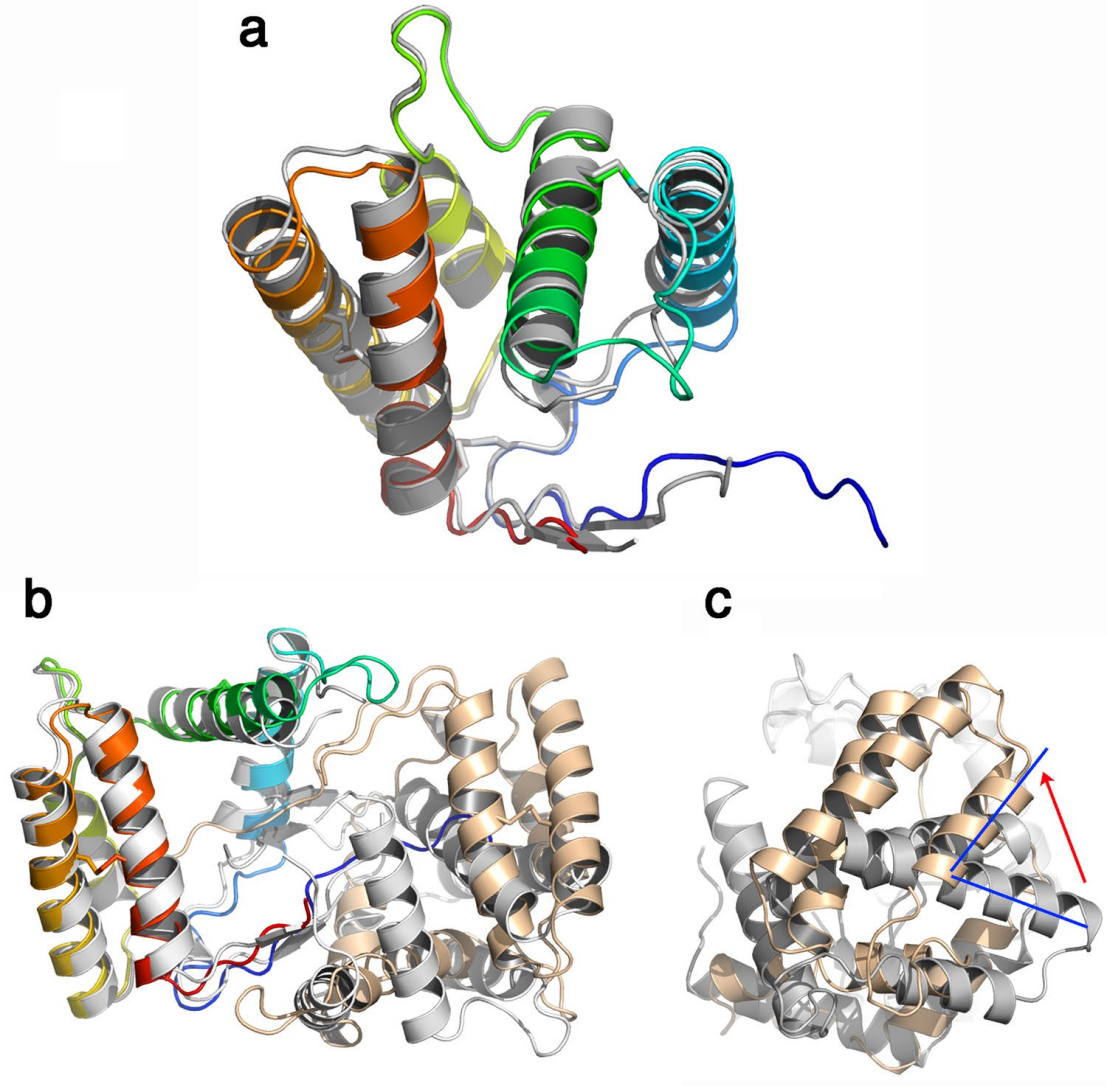
Comparison of crystal structures of VdesOBP1 forms P2_1_ and P3_2_21. (**a**) Ribbon view of the superposition of monomers belonging to forms P2_1_ (rainbow coloured) and P3_2_21 (grey). (**b**) Ribbon view of the crystallographic dimers of forms P2_1_ and P3_2_21. In form P2_1_ one monomer is rainbow coloured, the other one is coloured brown. The crystallographic dimer of P3_2_21 form is coloured light grey. One monomer of each form has been superimposed (left). The second monomers do not superimpose. B/same view rotated 90° around a vertical axis. Note the rotation of ~ 80° of the second monomer of form P3_2_21 relative to the second monomer of form P2_1_. Figure made with PyMOL (version 1.5.0.2. http://www.pymol.org).

The cloned protein is 147 residues long and possesses 6 cysteine residues (Fig. 4). The chain was built in the electron density map of the P2_1_ crystal form with the exception of residues 1–6 at the N-terminus, while the P3_2_21 crystal form starts at residue 11 and lacks loop 50–53. We checked the integrity of the protein in the crystal and our analysis showed that the protein was intact, confirming that the N-terminal segment is present but not ordered in the crystal. The r.m.s.d. value between the structures of the two VdesOBP1 crystal forms is 1.55 Å (Fig. 5a).

Being the structure best defined, our description will apply to the P2_1_ form. VdesOBP1 is mostly helical (Fig. 4). In VdesOBP1, five α-helices are observed in contrast with all classical insect OBP or PBPs (pheromone-binding proteins), such as BmorPBP^5^, Lush^23^ or LmadPBP^24^, which assemble six α-helices. The N- and C-terminal stretches are elongated forming a pseudo β-sheet of two strands (Fig. 4). Three disulphide bridges (21/141;C1–C6, 41/68;C2–C3, and 111/132;C4–C5, Fig. 4a) strengthen the 3D fold of VdesOBP1.

It has been reported that OBPs may form functional dimers^25–27^. However, it cannot be concluded firmly for or against dimerization of OBPs in their biological context. In the crystal, dimers have been observed in the case of AgamOBP1, but this observation may not apply to solutions, due to an insufficient contact area^26^. In the case of VdesOBP1, we observed a monomer using multi-angle static light scattering (MALS)/dynamic light scattering and RI coupled on-line with a size-exclusion chromatography (SEC) column (MALS/UV/RI/SEC) at three pH values (Supplementary Fig. S2 online). In contrast, the P2_1_ form contains two molecules in the asymmetric unit forming a crystallographic dimer (Fig. 4b). A crystallographic dimer can be reconstituted in the P3_2_21 form, using a symmetrical molecule in the crystal packing (Fig. 5b). However, the second monomer of one form does not display the same orientation relative to that of the second form when the first monomers are superimposed (Fig. 5b,c). Finally, the PISA server reported an ambiguous response for the P2_1_ form (score = 0.49) but discarded the possibility of a biological dimer in form P3_2_21 (score = 0). All in all, it seems highly probable that the biologically relevant form is a monomer.

### The binding crevice

**Figure 6:**
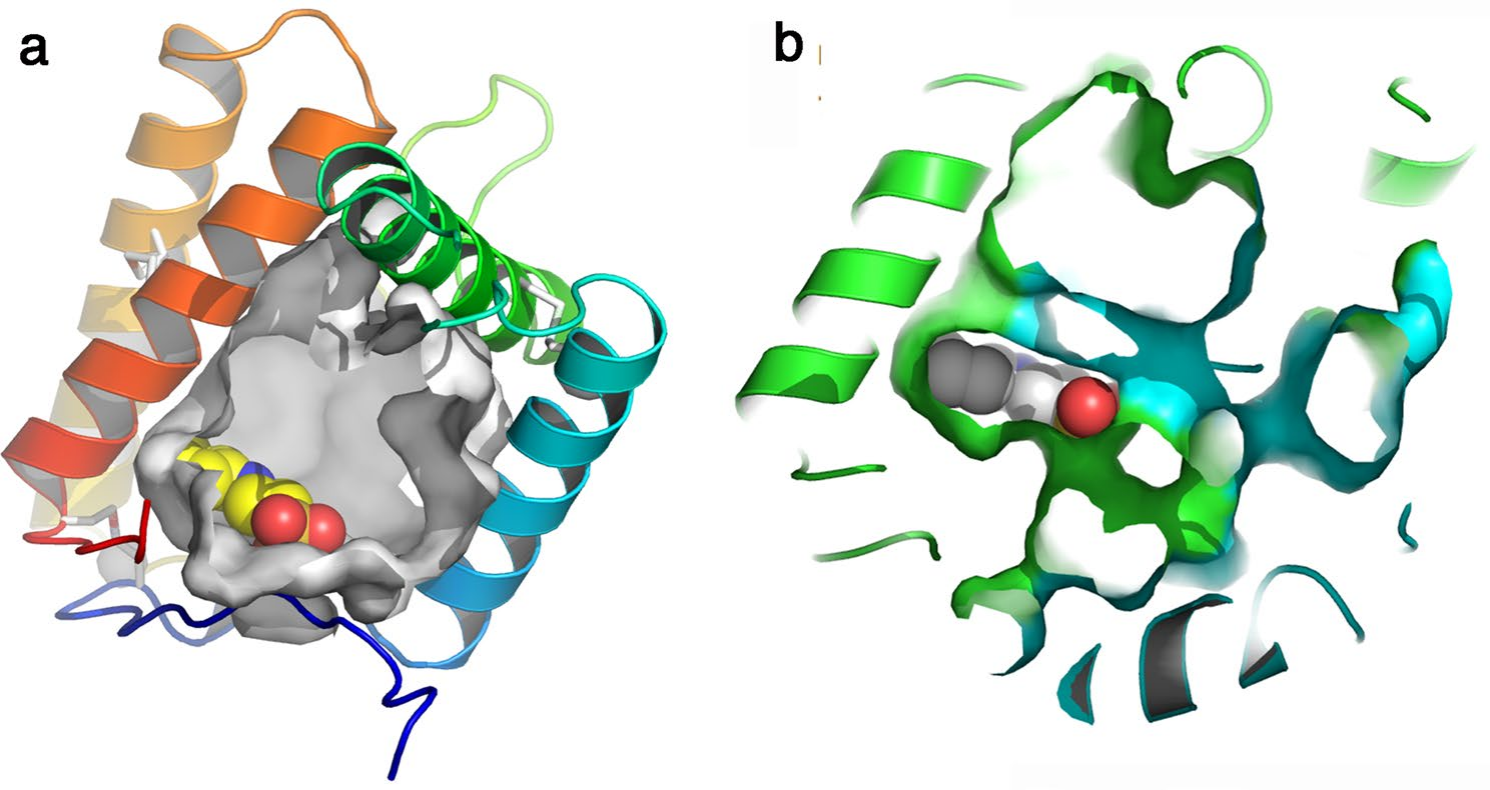
The internal cavity of VdesOBP1 form P2_1_. (**a**) Slabbed ribbon view of a monomer rainbow coloured. The cavity is represented by a grey surface and is wide open. The serendipitously bound buffer molecule 2-(N-cyclohexylamino)-ethane sulfonic acid (NCES) is displayed as atomic spheres (C: white, N: blue and O: red), and is stacked against the cavity wall. (**b**) Slabbed ribbon view of a crystallographic dimer rainbow coloured. The cavity is represented by a green surface originating from monomer 1 and a blue surface from monomer 2. The serendipitously bound buffer molecule NCES is displayed as atomic spheres (C: white, N: blue and O: red), and is stacked against the cavity walls from both monomers. Figure made with PyMOL (version 1.5.0.2. http://www.pymol.org).

The binding crevice, as detected by CavityPlus^28^, is large and wide open in the monomer, with dimensions of the floor of ~ 18 × 18 Å (Fig. 6a). This large surface implies that numerous amino acid side-chains form the walls of the binding site: hydrophobic side-chains (Pro14, Ile16, Trp18, Leu24, Pro 26, Val37, Val38, Leu70, Trp75, Tyr82, Ala87, Ile91, Tyr129, Met133, Ile137), polar non-charged residues (Thr66, Gln130) and charged residues (Glu30, Arg31, Lys34, Glu73, Glu90, Asp134). Hence, although most side chains are from hydrophobic/aromatic residues (16/24), two basic and four acidic residues complete the wall. The active site is empty, except in form P2_1_ where a buffer molecule, 2-(N-cyclohexylamino)-ethane sulfonic acid (NCES), is docked against a wall of the binding site (Fig. 6a). Examination of the binding site in the crystallographic dimer revealed that the N-terminus of each monomer protrudes deeply in the binding site of the other monomer, drastically reducing its size. This effect explains the serendipitous binding of NCES as this ligand interacts with the cell wall of one monomer and the C-terminal extension of the other one (Fig. 6b). The sulfonic acid moiety of NCES forms an ionic bond with Arg31A side chain (atom NH_2_) and with the main chain N–H of Lys12B.

### Comparison of VdesOBP1 with other OBPs/PBPs structures

**Figure 7:**
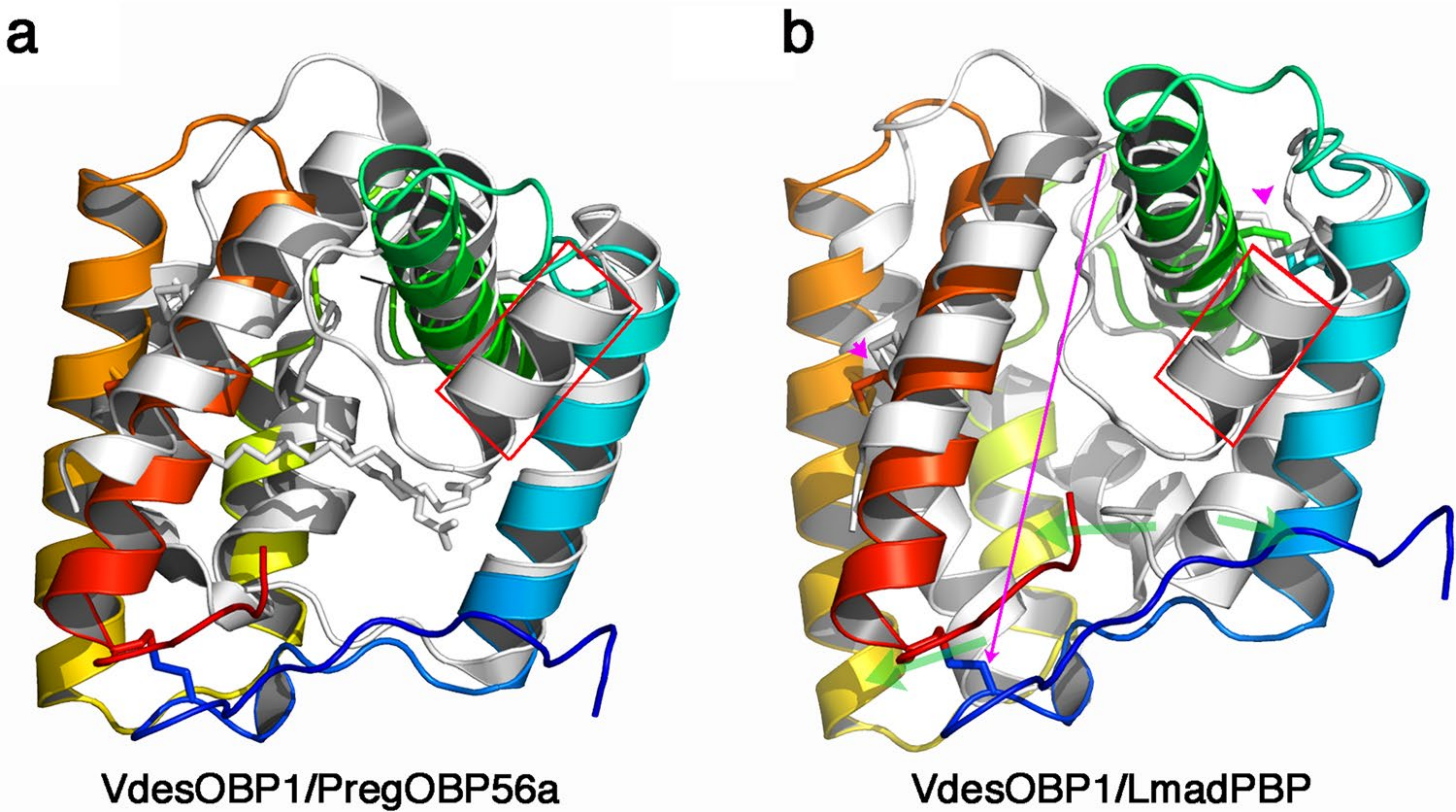
Comparison of crystal structures of VdesOBP1 with the closest related OBPs from *Phormia regina* (PregOBP56) and *Leucophera madereae* (LmadPBP). (**a**) Ribbon view of the superposition of monomers from VdesOBP1 (rainbow coloured) and PregOBP56 (light grey) with its two fatty acid ligands (grey sticks). Note the absence of the classical OBP fold helix 2 (red squared) in VdesOBP1. (**b**) Ribbon view of the superposition of monomers from VdesOBP1 (rainbow coloured) and LmadPBP. Note the absence of the classical OBP fold helix 2 (red squared) in VdesOBP1 as compared to LmadPBP (red squared) and the shift of one disulphide bridge (red arrow). The 3D positions of the two other disulphide bridges are conserved (purple arrow), in the final fold, although as result of different C1-C6, C2-C3, C4-C5 (VdesOBP1) and C1-C3, C2-C5, C4-C6 (LmadPBP) connectivities. Figure made with PyMOL (version 1.5.0.2. http://www.pymol.org).

A search on the PDB with the DALI server reported hits with good statistics, taking into account the Z-score and the r.m.s.d.^29^. The best hit is that of entry 5dic (Z = 7; r.m.s.d. 2.9 Å on 96/115 residues), the fatty acid binding protein OBP56a from the oral disk of the blowfly *Phormia regina* (PDB ID: 5dic, by Ishida, Y., Leal, W.S., Wilson, D.K., unpublished) with a non-classical OBP fold. This FBP/OBP has been shown to bind the C16 palmitic acid (but not the acetate), as well as the C18 stearic, oleic and linoleic acids, but not decanoic acid^30^. The structure is reported in complex with two molecules of palmitic acid in its wide cavity. Superposition of VdesOBP1 and PregOBP56a shows that VdesOBP1 helices 1, 2, 3, 4 and 5 superimpose well with PregOBP56a helices 1, 3, 4, 5 and 6. Helix 2 of PregOBP56a closes its binding cavity, while a similar effect is not observed in VdesOBP1. This PregOBP56a helix 2 is replaced in VdesOBP1 by a loop between helices 1 and 2, projected out of the protein core (Fig. 7a). However, the PregOBP56 disulphide bridges obey the C1–C3, C2–C5 and C4–C6 connectivity also present in classical OBPs, in contrast with VdesOBP1 (C1–C6, C2–C3, C4–C5). Another good fit (1ow4; Z = 5.4; r.m.s.d 3.3 Å on 8–90/120 residues) is observed with the PBP of the cockroach *Leucopheae maderae* (LmadPBP) (1ORG). In this case again, LmadPBP exhibits an extra helix instead of a loop between helices 1 and 2 in VdedOBP1 (Fig. 7b). Two disulphide bonds are conserved between the two proteins, the first bridging helices 1 and 2, the second bridging helices 4 and 5 of VdesOBP1 (Figs. 4a, 7b), although as result of different (C1–C6, C2–C3, C4–C5 in VdesOBP1 and C1–C3, C2–C5, C4–C6 in LmadPBP) cysteine linkages. The third disulphide bond that bridges helices 3 and 6 in LmadPBP is not observed in VdesOBP1. Instead, the third disulphide bridge is observed between helix 5 (corresponding to helix 6 in LmadPBP) and the extended N-terminus in VdesOBP1 (Fig. 7b).

### Ligand-binding assays

The purified VdesOBP1 did not show any affinity for either of the most common probes used in ligand-binding assays with OBPs, namely N-phenyl-1-naphthylamine (1-NPN) and 1-aminoanthracene (1-AMA). Extraction with dichloromethane to remove possible endogenous ligands did not prove effective. We also subjected the protein to a denaturing treatment with 8 M urea and 1 mM DTT, followed by slow renaturation by extensive dialysis, but we still could not observe any binding with either probes. We also tried 8-phenylamino-naphthalenesulfonic acid (ANS) as well as larger fluorescent reporters^31^, such as N-naphthyl-1-naphthylamine (1-NNN) and N-phenyl-1-aminoanthracene (PAA) with no effect.

**Figure 8:**
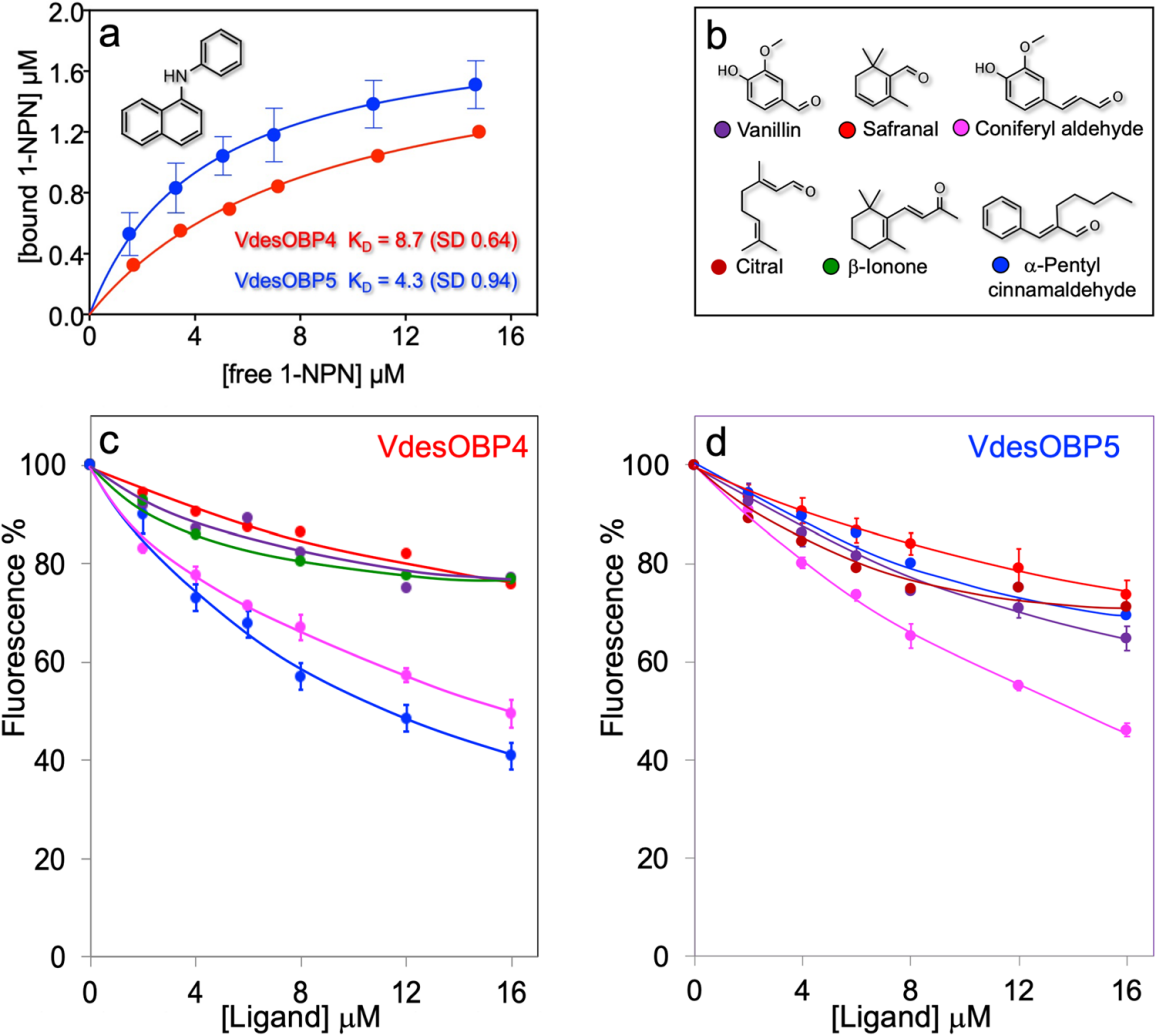
Ligand-binding assays. (**a**) Binding of the fluorescent probe N-phenylnaphthylamine (1-NPN) to VdesOBP4 and VdesOBP5. With the other two proteins expressed (VdesOBP1 and VdesOBP2), we could not observe the blue shift usually associated with binding of the fluorescent probe. (**b**) Structures of the ligand used in the competitive binding experiments. (**c,d**) Competitive binding assays. Only few chemicals were able to displace 1-NPN from the complex with VdesOBP4 and VdesOBP5. The same chemicals were also the best ligands for the other VdesOBPs (Supplementary Fig. S3 online).

It is still possible that the fluorescent probe binds the protein, but the event is not accompanied by a change in the emission spectrum. In fact, when adding 1-NPN to the protein, we observed a dose-dependent quenching of the intrinsic tryptophan fluorescence, suggestive of some affinity of this probe for VdesOBP1 (data not shown). In any case, competitive binding using a fluorescent reporter could not be used with VdesOBP1 or with VdesOBP2. Therefore, we attempted to find ligands for these two proteins monitoring the quenching of intrinsic tryptophan fluorescence. On the other hand, this method is limited to chemicals able to accept the energy transferred by the tryptophan (therefore structures with a region of conjugated π-electrons), and is not suitable to evaluate binding affinities. Instead, with VdesOBP4 and VdesOBP5 we obtained clear binding curves using 1-NPN as the fluorescent reporter (Fig. 8a). Therefore, we performed competitive binding experiments only with these two OBPs. Some of the displacement curves obtained with VdesOBP4 and VdesOBP5, using the ligands shown in Fig. 8b, are reported in Fig. 8c,d respectively. We can notice that only coniferyl aldehyde is able to decrease the 1-NPN fluorescence to 50% at the maximum concentration used, qualifying for a modest ligand. None of the other chemicals used in the experiments (listed in Supplementary Table S3 online) with the four proteins proved to be a better ligand. Representative curves relative to quenching of the intrinsic fluorescence of tryptophan in VdesOBP1 and VdesOBP2 are reported in Supplementary Fig. S3 online.

Summarising these data, we can first observe that some of the tested chemicals proved to be ligands for VdesOBP4 and VdesOBP5 in 1-NPN displacement assays, while they determined significant quenching of intrinsic tryptophan fluorescence in VdesOBP1 and VdesOBP2. The same few compounds (coniferyl aldehyde, safranal and vanillin) were the best ligands with all four proteins, although with poor dissociation constants between 10 and 20 μM. These ligands are associated with plants, in particular with leaf damage, and do not bear any relationship to the varroa habits. In particular, none of the honey bee pheromones, such as 9-ketodecanoic acid, homovanillyl alcohol or fatty acid esters were able to bind any of the tested proteins.

## Discussion

This work reports the original structural and functional characterization of four out of the five OBPs encoded in the genome of the mite *Varroa destructor*, a major pest for honey bee. Mass spectrometric analysis demonstrated that the six cysteines VdesOBPs, whose sequence position do not match the pattern in insect OBPs, are paired into three disulphide bridges (C1–C6, C2–C3 and C4–C5) different from that (C1–C3, C2–C5, C4–C6) of their insect counterparts. The overall folding, determined for VdesOBP1, is also novel and different from the conserved structure of insect OBPs, although some similar aspects can be recognised.

VdesOBP1 structure was determined in two crystal forms closely related. Its structure assembles 5 α-helices linked by three disulphide bridges. Comparison with classical OBPs reveals that the second of the six α-helices found in them is lacking in VdesOBP1. Although dimer formation of VdesOBP1 is observed in the crystal, it most probably does not represent the functional form in vivo. This dimerization is probably driven by the presence of a wide and fully open cavity, able to accommodate the extended N- and C-terminal stretches of the opposite monomer. The large cavity opening can be ascribed to the absence of helix 2 in OBPs classical fold, which restrains and closes the internal binding site. In VdesOBP1, this helix is absent and replaced by a loop projected outside the protein core. Two disulphide bridges are conserved as compared to classical OBPs, although associated with different sequential cysteine linkages. These observations suggest that VdesOBP1 possesses an original fold remotely related to classical OBPs. Comparison with PregOBP56a suggests that two to three fatty acids could bind inside the VdesOBP1 cavity. Such an arrangement of three ligands was observed in *Mamestra brassicae* CSP (CSPMbraA6)^30^.

The unique structure of VdesOBP1 and the lack of strong ligands pose questions that, at this stage, can only be addressed by formulating hypotheses and models to be verified by further experimental work. A first idea, based on the limited binding activity of these proteins and on the small number of genes (4–5) expressed in *V. destructor*, as well as in other mites and ticks, might suggest that these genes represent by-products of evolution, which are not used any more. In fact, at least two more families of carrier proteins could be active in Chelicerata, namely NPC2 proteins and lipocalins, apart from a third family (CCPs: candidate carrier proteins) that so far seems to be specific of spiders^21^. NPCs and lipocalins are represented in varroa by 6 and 9 members, respectively, while in other Chelicerata often 10–20 members of NPC2s have been identified, with exceptions of nearly 50 genes, as in the spider mite *Tetranychus urticae*. Although making sense for the reason reported above, the idea that OBPs in Chelicerata might represent a dead end in evolution and a failed attempt to make efficient carrier proteins contrasts with proteomic findings showing significant expression of such proteins^22^. In fact, four (VdesOBP1-4) of the five VdesOBPs were identified in different parts of *V. destructor*, with one of them (VdesOBP4) particularly abundant in sensory organs, namely the first pair of legs and the mouthparts.

If then the expression of OBPs at the protein level indicates that they are functional, then, along with a second hypothesis, their poor affinity might be related to the absence of post-translational modifications. It has been recently shown that the pig OBP1 can bind fatty acids or steroids depending on the phosphorylation and glycosylation status of the protein^32^. Our varroa OBPs, being expressed in bacteria, are devoid of any post-translational modification. On the other hand, we do not have any information on possible phosphorylation and/or glycosylation of these proteins in their native forms. This is certainly an interesting aspect, although not easy to investigate, not only for the tiny size of sensory organs, but also because phosphorylation, in particular, may be present only in certain time windows related to the physiology and the behaviour of the mite. Indeed, protein phosphorylation is highly related to signal transduction events regulating essential physiological process, and it has been reported to drastically modify the binding properties of OBPs^32^. Thus, we searched for putative phosphorylation sites in the five OBP sequences, and we found a very large number of serine and threonine residues that could be potentially phosphorylated (Supplementary Fig. S4 online). In addition, we found few tyrosine residues as potential phosphorylation sites (2 in VdesOBP1, 1 in VdesOBP2 and VdesOBP3, 4 in VdesOBP5).

In the three-dimensional structure of VdesOBP1, no serine or threonine residues are found inside the binding pocket. However, phosphorylation of serine and threonine residues outside the binding pocket (as well as glycosylation of the protein) can affect the folding of the protein and influence its binding affinities. Further speculation would be inappropriate based on the available experimental data. Certainly, a structural study of the native proteins, regarding their post-translational modifications would be essential before proposing alternative modes of action.

## Materials and methods

### Biological material

*V. destructor* mites were collected from the cells of an infected honeybee hive and used immediately for RNA extraction.

### Reagents

Chemical compounds for buffers and solutions, as well as ligands for binding assays were of analytical grade from Merck, Austria. Ligands were dissolved in spectroscopic grade (Uvasol) methanol. Oligonucleotides and synthetic genes were custom synthesised at Eurofins Genomics, Germany. Enzymes and kits for DNA extraction and purification were from New England Biolabs, USA.

### RNA extraction and gene cloning

Total RNA was extracted using Tri-reagent (Sigma-Aldrich) according to the protocol provided. cDNA was prepared from 2 μL of total RNA using the kit qScript cDNA SuperMix (Quanta Bio) and the manufacturer’s procedure. For sequence verification, PCR was performed on cDNA using specific primers at the 5’ end and at the 3’ end. The primers, which were also used for cloning into the expression vector, also contained the restriction sites NdeI and EcoRI at the 5’ and the 3’ ends, respectively. After digestion, the PCR products were inserted into pET30 vector previously linearized with the same enzymes. This procedure was adopted for all the genes of *V. destructor*, except for VdesOBP1 used for structural studies, which was produced from a synthetic gene (Eurofins Genomics), whose nucleotide sequence was optimized for *E. coli* expression.

### Gene cloning

Synthetic genes encoding VdesOBP1 and VdesOBP2 were custom synthesised at Eurofins Genomics, Germany, and optimised for *E. coli* expression. After digestion with restriction enzymes Nde I and Eco RI, the genes were subcloned into the pET-30 expression vector (Novagen, Darmstadt, Germany). The sequences encoding VdesOBP4 and VdesOBP5, instead, were amplified directly from cDNA, digested and cloned into the pET-30. The constructs encoded only the mature protein sequences preceded by an additional methionine, which is not considered here for amino acid numbering. Expression was performed in *E. coli* BL-21 (DE3) competent cells. The sequences of the primers used are reported in Supplementary Table S4 online.

### Protein expression and purification

Protein synthesis was induced by adding IPTG (to a final concentration of 0.4 mM) to the *E. coli* culture, grown for two additional hours at 37 °C, then harvested by centrifugation, sonicated in 50 mM Tris/HCl pH 7.4 and centrifuged at high speed. The VdesOBPs were obtained in good yield in the supernatant, except for VdesOBP4, which was produced in poor yield and was present in the pellet, from which it was solubilized with 8 M urea and 1 mM DTT. Refolding was obtained by simple dialysis for three days (with changes of buffer every day) against 50 mM Tris–HCl, pH 7.4^25^. All VdesOBPs were purified by 2–3 chromatographic steps on anion-exchange columns DE-52 and HiPrep-Q 16/10, 20 mL (Bio-Rad). The purification process was monitored at each step by polyacrylamide gel electrophoresis in denaturing conditions (SDS-PAGE).

### Protein alkylation and digestion, and mass spectrometry analysis

Samples of *V. destructor* OBP1, OBP2 and OBP5 (20–50 μg) dissolved in 0.1 M tetraethylammonium bicarbonate (TEAB), pH 6.5, containing 4 M guanidinium chloride, were treated with iodoacetamide (0.5 M final concentration) for 30 min, in the dark; then, proteins were insolubilized by addition of 6 vol of cold acetone, leaving the samples at -20 °C, overnight. After centrifugation at 12,000 rpm at 4° C, for 20 min, supernatants were removed, and recovered pellets were dried with a SpeedVac device (ThermoFisher Scientific, USA). Recovered proteins were dissolved in 0.05 M TEAB, pH 6.5 (2 µg/μL final concentration), treated with trypsin (1:10 w/w enzyme/substrate) for 16 h, at 37 °C, then with chymotrypsin (1:8 w/w enzyme/substrate) for 16 h, at 37 °C. Protein digests were desalted with ZipTip C18 (Millipore, USA) and directly analyzed with a UltiMate 3000 HPLC RSLC nano-chromatographer (ThermoFisher Scientific) linked *on-line* through a nano-Spray ion source (Thermo Fisher Scientific) to a Q-ExactivePlus mass spectrometer (Thermo Fisher Scientific)^33^. Peptides were separated on an Acclaim PepMap RSLC C18 column (150 mm × 75 μm ID; 2 μm particle size; 100 Å pore size) (Thermo Fisher Scientific), at a flow rate of 300 nL/min, using a gradient of solvent B (19.92/80/0.08 v/v/v water/acetonitrile/formic acid) in solvent A (99.9/0.1 v/v water/formic acid). Solvent B started at 3%, increased linearly to 40% in 45 min, then raised to 80% in 5 min, where it remained for additional 4 min, before rapidly returning to 3%. Mass spectrometer worked in positive polarity using a data-dependent mode, performing a full MS1 scan in the range *m/z* 345–1350, at a nominal resolution of 70,000, followed by MS/MS scans of the 10 most abundant ions in high energy collisional dissociation mode^34^. MS/MS spectra were acquired in a dynamic *m/z* range, with a nominal resolution of 17,500, a normalized collision energy of 28%, an automatic gain control target of 50,000, a maximum ion injection time of 110 ms, an isolation window of 1.2 m*/z* and a dynamic exclusion setting of 20 s.

### Bioinformatics for peptide identification

Firstly, raw mass data files were analyzed with Proteome Discoverer v. 2.4 package (Thermo Fisher Scientific), running by Mascot v. 2.6.1 (Matrix Science, UK) and Byonic v. 2.6 (Protein Metrics, USA) software. Database searching was performed against a database containing the sequences of *V. destructor* OBP1, OBP2 and OBP5, trypsin, chymotrypsin and common protein contaminants. Parameters for database searching were variable oxidation at Met, deamidation at Asn/Gln, pyroglutamate formation at Gln and carbamidomethylation at Cys. Mass tolerance setting included ± 10 ppm for precursor ions and ± 0.05 Da for fragmentation ions^35^. Proteolytic enzyme and maximum number of missed cleavages were set to trypsin, chymotrypsin and 5, respectively. Proteome Discoverer peptide candidates were considered confidently identified only when the following criteria were satisfied: (1) protein and peptide false discovery rate (FDR) confidence: high; (2) peptide Mascot score: > 30; (3) peptide spectrum matches (PSMs): unambiguous; (4) peptide rank (rank of the peptide match): 1; (5) Delta CN (normalized score difference between the selected PSM and the highest-scoring PSM for that spectrum): 0. Byonic peptide candidates were considered confidently identified only when the following criteria were satisfied: (1) PEP 2D and PEP 1D: < 10 × 10^−5^; (2) FDR: 0; (3) q-value 2D and q-value 1D: < 10 × 10^−5^. Finally, disulphide bridge assignment was obtained using dedicated BioPharma Finder v. 4.0 (Thermo Fisher Scientific) and pLink v. 2.3.9 software^36^. Both programs were used for database searching, enabling the specific function of disulphide-linked peptides attribution, and applying the additional settings reported above for Proteome Discoverer and Byonic examinations. Disulphide-bridged peptide identification was considered reliable when BioPharma Finder and pLink results showed a “confidence score” > 95 and/or an “E-value” < 1.0^–10^, respectively. Manual interpretation and verification of the candidate spectra were always performed.

### Crystallization, data collection and structure determination of the P3_2_21 form

The protein was concentrated to 20 mg/ml. Screening experiments were performed using several commercial kits. The sitting-drop method in Swissci plates was used. The reservoirs of the Swcissi Plates were filled using a Genesis II (TECAN) pipetting robot. The nanodrops were dispensed by a MOSQUITO Crystal (TTP Labtech). Respectively 300 nL, 200 nL and 100 nL of protein were dispensed onto the central of three-sitting-drop shelves. 100 nL of the reservoir solution were added to the protein drops. The plates were sealed with a transparent film and stored in a R1000 crystallization hotel (Formulatrix). Small crystals were obtained in 0.2 M cadmium chloride, 2.2 M ammonium sulfate. Those crystals were used for micro-seeding. The sitting-drop method in Greiner plates was used and new crystals were obtained by doing a re-screen in the commercial screen PEGII with the initial condition in which the first crystals were obtained. The droplet with the thicker crystals contained 2/3 of the initial condition (0.2 M cadmium chloride, 2.2 M ammonium sulfate) + 1/3 of PEG II screen (0.2 M calcium chloride, 0.1 M HEPES pH 7.5; 30% w/v PEG 4000).

The crystal was cryo-cooled at 100 °K within this crystallization liquor, with the addition of 10% glycerol as cryoprotectant. Structure determination used SAD-phasing method at remote wavelengths (λ = 1.51200 Å) with sulphur atom anomalous dispersion on native crystals. A strong theoretical Bijvoet ratio of 1.12% was expected from the 10 sulphur atoms of the protein that contribute to the anomalous signal at wavelength of 1.512 Å. The sulphur-SAD data set was collected on the PROXIMA-2 beamline (SOLEIL) equipped with a high performance micro-diffractometer with a mini-kappa (MD2), an X-ray fluorescence detector (KETEK), and a fast, low-noise, photon-counting, area detector EIGER X 9 M (238 fps in 9 M mode, 750 fps in 4 M mode). A redundant data set was collected at 1.97 Å resolution. including 3600 images of 0.1° rotation collected with four K orientations of 0°, 30°, 45° and 60°. The data set was processed with XDS^37^. The data sets were indexed and integrated with Pointless and scaled with CCP4 SCALA^38^. VdesOBP1 crystallized in space group P3_2_21 with cell dimensions a = 53.84 Å, b = 53.84 Å, and c = 91.74 Å and and γ = 120 with one molecule in the asymmetric unit, corresponding to a solvent content of 50.87%. Six sulfur atom sites were found by using PHENIX Autosol^39^. The results from PHENIX include a PDB containing the anomalous scatterers, a MTZ file containing the experimentally determined phases, and the optimized phases from density modification. Structure initial construction was performed with PHENIX Autosol^39^ and was not further refined. A second data set was collected at 1.20 Å resolution at a wavelength of 0.97856 Å. The ab-initio determined structure was used as a starting model for model refinement. Refinement was performed with REFMAC5^40^ and AutoBUSTER^41^ alternated with rebuilding using COOT^42^.

### Crystallization, data collection and structure determination of the P2_1_ form

The protein at 20 mg/mL was subjected to screening experiments that were performed using several commercial kits. VdesOBP1 was set in 2/3 of the initial crystallization conditions (0.1 M CHES pH 9.5, , 30% w/v PEG3000) and 1/3 of ammonium sulfate screen (0.2 M sodium nitrate, 2.2 M ammonium sulfate). Crystals of VdesOBP1 were briefly soaked in crystallization solution supplemented with 10% (v/v) polyethylenglycol. Diffraction data were collected at 1.2 Å resolution on beamline Proxima-1 at SOLEIL, Paris, France. The data sets were integrated with XDS^37^ and were scaled with CCP4 SCALA^38^. VdesOBP1 crystallized in space group P2_1_ with cell dimensions a = 31.6 Å, b = 112.96 Å, c = 41.82 Å and β = 98.95, with two molecules in the asymmetric unit, corresponding to a solvent content of 44.48%. The structure was solved by molecular replacement with MOLREP^43^ using the structure of VdesOBP1 form P3_2_21 as starting model. Refinement was performed with REFMAC5^40^ and AutoBUSTER^41^, and the structures were corrected with COOT^42^. Figures 4, 5, 6 and 7 were made with PyMOL (version 1.5.0.2. http://www.pymol.org).

### Multi-angle light scattering

100 μL of purified VdesOBP1 were analyzed by SEC-MALS using a HPLC system (Thermo Scientific) equipped with a Superdex 75 column coupled with a multi-angle light scattering device (Dawn 8, Wyatt Technology) and an Optilab TrEX differential refractive index detector (Optilab TrEX, Wyatt Technology). At first, the column was first equilibrated with a buffer containing 0.02% w/v NaN_3_ for several hours, until the baseline of the Optilab TrEX signal was stable. Then, light scattering and concentration data were analysed in ASTRA to determine molecular weight and size distribution. Inter-detector band broadening and alignment were applied using the algorithms in ASTRA 7 (Wyatt Technology, USA) using BSA protein standard according to manufacturer’s recommended methods. Three different buffers were used to understand the behaviour of the protein at different pH values, namely 50 mM CHES pH 10, 50 mM Hepes pH 7 and 50 mM sodium acetate pH 5. The temperature-controlled autosampler was kept at 10 °C.

### Ligand-binding assays

Direct binding of fluorescent probes was monitored by adding aliquots of 1 mM methanol solution of the probe to a 2 μM solution of the protein in 50 mM Tris–HCl, pH 7.4 to final concentrations of 2 to 16 μM. The excitation wavelength was 337 nm and intensities were measured on the maximum of the peak, usually between around 410 and 420 nm. For VdesOBP1 and VdesOBP2, binding of ligands was evaluated by monitoring the intrinsic tryptophan quenching of the protein. Excitation wavelength was 295 nm and intensities were recorded around 335–340 nm. For VdesOBP4 and VdesOBP5, competitive binding experiments were performed by titrating a solution of protein and 1-NPN both at 2 μM in Tris–HCl buffer, pH 7.4 with 1 mM methanolic solutions of ligands to final concentrations of 2–16 μM. All measurements were performed with a FL 6500 spectrofluorometer (PerkinElmer); slits were set at 5 nm for both excitation and emission, and 1 cm path quartz cuvettes were used.

Dissociation constants for 1-NPN were evaluated using Prism software. Affinities of other ligands were calculated from the corresponding [IC]_50_ values (the concentration of each ligand halving the initial value of fluorescence), from the equation:

$${\text{K}}_{{\text{d}}} = \left[ {{\text{IC}}} \right]_{{50}} /1 + \left[ {1 - {\text{NPN}}} \right]/{\text{K}}_{{{\text{NPN}}}}$$

where [1-NPN] is the concentration of free 1-NPN and K_NPN_ is the dissociation constant of the complex OBP/1-NPN.

## Supplementary Information

Supplementary Information.

## Data Availability

The three-dimensional structure of VdesOBP1 reported in this paper has been deposited in the database with PDB ID 7NYJ and 7NZA.
