# Supplementary material for: A new non-classical fold of varroa odorant-binding proteins reveals a wide open internal cavity
Source: Sci Rep. 2021 Jun 23;11:13172. doi: 10.1038/s41598-021-92604-2 (PMC8222343; doi:10.1038/s41598-021-92604-2)
Supplement: Supplementary file 1 — Supplementary Information. [file 41598_2021_92604_MOESM1_ESM.pdf]

**Supplementary Material**

**A new non-classical fold of varroa odorant-binding proteins reveals a wide open internal cavity**

Beatrice Amigues[1#], Jiao Zhu[2,4#], Anais Gaubert[1], Simona Arena[3], Giovanni Renzone[3], Philippe Leone[1], Isabella Maria Fischer[2], Harald Paulsen[4], Wolfgang Knoll[2,5], Andrea Scaloni[3], Alain Roussel[1], Christian Cambillau[1]*, Paolo Pelosi[2]*

[1]*Architecture et Fonction des Macromolécules Biologiques (AFMB, UMR 6098), Centre National de la Recherche Scientifique (CNRS) & Aix-Marseille Université (AMU), Campus de Luminy, Case 932, 13288 Marseille Cedex 09, France*

[2]*Austrian Institute of Technology GmbH, Biosensor Technologies, Konrad-Lorenz Straße, 24, 3430 Tulln, Austria*

[3]*Proteomics & Mass Spectrometry Laboratory, ISPAAM, National Research Council, 80147 Napoli, Italy.*

[4]*Faculty of Biology, Institute of Molecular Physiology, Johannes Gutenberg-Universität, 55099 Mainz, Germany*

[5]*Department of Physics and Chemistry of Materials, Faculty of Medicine/Dental Medicine,*

*Danube Private University, Krems, Austria*

**Supplementary Table S1. Sequences of ticks and mites used for the phylogenetic tree reported in Fig. 1**

```
>VdesOBP1_XP_022672530 .1 uncharacterized protein LOC111255133 [Varroa destructor]
MKVFVIIALVGMAKAAPQAPASATPAKVPVIEWGKCEQLKPSESERTSKAAVVDKCLQSLPLPDPEKATQQEIDKHRESVTTCALKAEGWFDDEGVYKFDRARNEIKNKKLDSEVEEAVLLKHDACQKEATEKH
DDYINQVQLYQACMDYNISQICGIKVMV
>VdesOBP2_XP_022645714 .1 uncharacterized protein LOC111243821 [Varroa destructor]
MKTFIGLVLVGMAMAAPPASGGPEAGKPPTIEWGKCSQLKPSDSERMTKAAVVEKCVQSLPLPEAGKASQAEIEKHREDVTTCALKAEGWFDDKGVYKFDRARDEIKNKKLASDIETPVLEKHAECQKEAGEKH
TSDYIKQVQLYQACMDFNISQICGIKVVS
>VdesOBP3_XP_022653293 .1 uncharacterized protein LOC111247029 [Varroa destructor]
MNAAVYIVTAMLACCCSADTASGDHASDTSQFGKWRTCLADKLPAKKREVFDECFIKPGGTDMNKFRRGLNCVLTSDGLLKNHKVDLKKMSMTAASAGSPELKKAYENCPKDDKNTSEDRSIKCLIDHLEVDCK
LASK
>VdesOBP4_XP_022653281 .1 uncharacterized protein LOC111247024 [Varroa destructor]
MYENRLLHLNGICGGGIQADRCEPAYLPDTVVRIIVKMKTLVVALLASTACLTAADTATGDHQPDSNQFGRWRTCLKSKIPENKQPGMEACINKPGGTDMSKFRRGLGCVLESYGIVNNHRVDLTKMRSSASLV
TSPELKKAFMECPKDDKNSSLDRSIKCVIDHLETSCSAVKAQG
>VdesOBP5_XP_022666940 .1 uncharacterized protein LOC111252778 [Varroa destructor]
MHRSFVLQCCGLLFIVGLALAGPLAKDKSERITPKQTVEEFFTSNWGECEPLKNPLVLPEISDAATWERCRVHIPLSPDQVEEALPNLSRCMLKVIGWVRANGNMNLTKYMDYLSQAGLDDKTLELTKTAHEKC
SQRALSDGSGDYKEQDKIYVECIFRHFDKTCPPNFQAAVKQGYIVGITQISFVPRYDEVEVESDTDAETEGQAGPSVADATGEAAANDNANGSGGPQ

>Turt_XP_015786166 .1 uncharacterized protein LOC107363453 [Tetranychus urticae]
MKNLQSITILCLIFGFIVSTVYSQPLGSSDSNPDTQDTKEASTSVNDKSSDQEGSSESDEDAPTPVAGPHRPPTINWGKCPQLEPKEAEKKQKADIIRTCLKSITLPENITQESVEKHRTEVAKCALNAENWFA
EDGSYRYDKAESEIKNKKLQPEIEPQIVNQHSECRKEAEEQFPSGIAQIQLYQACMDYHISMICGIQIVGTGYSQ
>Turt_XP_015794342 .1 uncharacterized protein LOC107370892 [Tetranychus urticae]
MNYLSLCVFIIFFLTIGFSESARLPRSTSSPATSSSTTTTSSTVNNKKNEENQPSTSSASSSAGSATGGPTINWGRCPQLEPTNEEKIKKAAVITKCLESTPVPVNITRETVELHREQVAACALRMEGWFNGQG
LYRFDKAENEIKAKKLTREVEEKVLSFHRQCKDEAEEKYPVSSNQLIAQIQLYQACMDYFISEVCGIEVQIAGSEGSSE
>Turt_XP_015782945 .1 uncharacterized protein LOC107360741 [Tetranychus urticae]
MFLISLCLIVLVVNSSNCAPQATPNRQGGFSGQLQGPPQPPQINWGKCPQLEPVESDKKAKADIISSCLKSIALPENMTQETVEKHRTEVARCALNAENWFTEDGKYRYDKAESEIKAKKLSQEVEPQILRQHG
ECRKEAEGQFPSGIAQIQLYQACMDYHISQICGITIVGPSSPQ
>Turt_XP_015791022 .1 uncharacterized protein LOC107367785 [Tetranychus urticae]
MILITLCLVVLAINSSYGQPQRFLAPQPTSDQLEGSPQGGFPGQPQGPPQPPQINWGKCPQLEPKESDKKAKADIISSCIKSIPLPENVTQESIEKHRTEVAICALNAENWFSADMKYRYDKAESEIRAKKLVS
DVEPLVLRQHGECRKEAEGQFPSGIAQVQLYQACMDYHISQICGITIVGASSPQ

>Hqin_AAX37829 .1 Hq05 [Haemaphysalis qinghaiensis]
MVSLKAVLFVCALAFFTCVGSAPAAEPKQPEVNWGKCPQLKPTEEEKQQKAQVVDQCLDKIPIPDLDTANETVILKHREDVTTCALKTEGWFNKNGHYRFDRAKTEISNKKLPADVQPQVIAKHDECKKEAETK
FPKNFIAQVQLYQACMDHHISQLCGVKIEGDSHGGHSAGGKKASA
>Hfla_AYH64978 .1 immunogenic protein [Haemaphysalis flava]
MVAFKAALFICALALPVYAQNAPAAEPQRPEINWGKCPQLQPSKEERQQKALVIDTCLEKVPLPDVEHANETVIQQHREDVTTCALHSEGWFNKNGQYRFDRARTEILNKKLAAEVEPQVLAKHDECKKEAEEK
FAHQFVAQVQLYQACMDYHISQICGIQIQALPQAGAAPPAQG
>Hlon_ADK47399.1 immunogenic protein [Haemaphysalis longicornis]
MVAFKAALFICALALPVYAQNAPAAEPQRPDINWGKCPQLQPSKEERQQKALAIDTCLEKVPLPDVEHANETVIQQHREDVTTCALHSEGWFNKNGQYRFDRARTEILNKKLAAEVEPQVLAKHDECKKEAEEK
FAHQFVAQVQLYQACMDYHISQICGIQIQAGAPAGGVPQQPPQQG

>Isca_XP_002399632 .1 uncharacterized protein LOC8038114 [Ixodes scapularis]
MVAFKVSVAVLLCVGSAFAIPAGGKDLDAAAQTQAGVPPRPQINWGKCPQLQPSENERQQKALVIDQCLDKVPLPDVDTANDTVIQQHRQDVTTCALKTEGWFNSNGQYRFDRARTEILNKKLAKDVEPQVLSK
HDECKKEAEEKFAKNFVAQVQLYQACMDYHISQICGIQIAQAAEAHPQGAQQG
>Isca_EEC12103 .1 hypothetical protein IscW_ISCW008703 [Ixodes scapularis]
MWTSAGKSALVTLGVLLAVGVGLGDAGPVDPKKTDKPEKQTIHEFFSSNWGACEPLKNPLVIPEMSNPTTWVKCQKHLPEHPQDIEEYLPRLSRCMLKSTGWVRSNGNMNLSKYLQYLSLLGLDDETMNGTREA
HKGCIAKAYKEKDYKTQDKVYVECIFKFFTKSCSANFQGAVKEGYIVGITQLSFMPRMGPLEVVDDVEAEVSEDDAPQDVLPVTNSTSTS
>Isca_XP_002433530 .1 uncharacterized protein LOC8051706 [Ixodes scapularis]
MKILLITVVVCVFAATDASRRSTRRQQFTDWRNCMVEQLPSEKKPIYDACYNRSRGTDMHKFREGLKCVLTDYNLLNGTKIDLAAMTTRASSVSGKLGQAFTDCPKDDHNRNAAKGVKCIIDRLETDCPVPTGS
TAH

>Gocc_XP_003747300 .1 uncharacterized protein LOC100900705 [Galendromus occidentalis]
MKIFFAVALTILVCEVSADTASGDHTSDDSKFGKWRSCLASKLPADKQKVHEDCFKKPGGTDMSKFRRGLSCVLDSYGVVKDHKIDQAKMASTATSITSPELKKAFTECPKDDKNVSEDRTIKCVIDNLETTCK
LST
>Gocc_XP_003742126 .1 uncharacterized protein LOC100907366 [Galendromus occidentalis]
MNTFSCVLLGVLVAGACAGPIAKDKSERISPKQTVEEFFTSNWGSCEPLKNPLVLPEISDASTWERCRAHIPISPEQVEEALPNLSRCMLKVIGWVRPNGNMNLSKYMDYLSQAGLDGKTLELTKIAHEKCSSR
AFSQAANDYKEQDKIYVECIFRHFDKTCPPNFQAAVKQGYIVGITQISFVPRYDEVDEAGPVEEDAEPQQTQQEDSNSSIKDSDAQ
>Gocc-2_XP_003741537 .1 uncharacterized protein LOC100907454 [Galendromus occidentalis]
MKVFVLAAFVGLALAAPQAQQGASGGAPKNPIIEWGKCNQLKPSEGERTSKAAVVDKCLQQLPLPDPEKASQAEIDKHRESVTTCALKAEGWFDDKGVYKFDRARNEIKNKKLDSEIESQVLVKHDECQKEATE
KHTDFINQVQLYQACMDYNISQICGIKVMV

>Tmer_OQR79252 .1 hypothetical protein BIW11_05867 [Tropilaelaps mercedesae]
MKTLVIALIAGTACLAAADIATGDHQPDTNQFGQWRTCLKSKIPQDKQATMDNCVKKPGGTDMAKFRRGISCVLESYGVVNGHRVDLRKMGSTASSVRSPELKKAFQECPKDDKNASLDRSIKCVIDHLETSCP
LVKAQG
>Tmer_OQR80083 .1 hypothetical protein BIW11_00054, partial [Tropilaelaps mercedesae]
VEQFFTSNWGECEPLKNPLVLPEISDGSTWERCRAHIPLTPEQVEEALPNLSRCMLKVIGWVRPNGNMNLTKYMDYLSQAGLDEKTLELTKIAHEKCSQRALSDGAGNYKEQDKIYVECIFRHFDKTCPPNFQA
AVKQGYIVGITQISFVPRYDDVEMEVDSDADAEDPNGAYPGPGADDANASSGGSSNNNNKDDSNNNNKADSNGDAQ
>Tmer_OQR73552 .1 hypothetical protein BIW11_09656 [Tropilaelaps mercedesae]
```

MKVFVVMALMGMAMAAPQAPAAPTSAKAPVIEWGKCDQLKPSENERTSKAAVVDKCLQSLPLPDPEKATQQEIDKHRESVTTCALKAEGWFDDKGVYKFDRARNEIKNKKLDGEIEAAVLVKHDACQKEATEKH
EDYINQVQLYQACMDYNISQICGIKVMV

>Rann_ABV53333 .1 unknown larval protein [Rhipicephalus annulatus]
MVAFKAALILCVLVLPAYAQTAGAEPPRPEINWGKCPQLQPSKEERQQKALVIDTCLEKVPLPDVEHANETVIQQHREDVTTCALHSEGWFNKNGQYRFDRARTEILNKKLAADVEPKVLAKHDECKKEAEEKF
AHQFVAQVQLYQACMDYHISQICGIQIQGGAGAPAHG
>Rsan_AQP25670 .1 Rs 05br antigen [Rhipicephalus sanguineus]
MVAFKAALILCVLVLPAYAQTAGAEPPRPEINWGKCPQLQPSKEERQQKALVIDTCLEKVPLPDVEHANETVIQQHREDVTTCALHSEGWFNKNGQYRFDRARTEILNKKLAADVEPKVLAKHDECKKEAEEKF
AHQFVAQVQLYQACMDYHISQICGIQIQGAGGAGAPAHG

>Ldel_RWS22631 .1 hypothetical protein B4U80_07214, partial [Leptotrombidium deliense]
PPQQGQQFHPQQQGPPQQQQGPGGEQPPYPGSPQIYWGRCPQLEPTMDEKVKKAAVITKCLETTPVPQNITQESVEVHREQVAACALQMEGWFNDKGLYKYDKAESEIKAKRLSKEIEERILSYHKQCKEEAEE
KFPVSNKALISQIQLYQACMDYFISDVCGIDVLRR
>Ldel_RWS28635 -.1 hypothetical protein B4U80_06376 [Leptotrombidium deliense]
MLKTVCFLLFAIAAVKCQQQPPEAPQPVPQAPPQPPPQPRINWGQCPQLEPKESDKQAKAKVIQGCLQKIPVPTNITQETIVQHQREVALCALTTEQWFNEQGKYRYEKAENEIKRKGLQPVIQTRIVFHHNKC
QTEAKEKQDVIQEVQMYQACMDYYIAQICGITVTTQ

>Dtin_RWS02013 .1 hypothetical protein B4U79_11820 [Dinothrombium tinctorium]
MWKTVAIVIFFCVLFVDCQQPQEPKINWGKCSKIEPTEQDKQEKAKVIQGCLKQVPLPKNVTRESVAQHQREVAVCALNAENWFNEEGKYRYEKAERQIKSKKLEKEIETKILFHHNKCKREAQEKTEIIEEVQ
LYQACMDYYIAQICEITVN
>Dtin_RWS11187 .1 hypothetical protein B4U79_15176 [Dinothrombium tinctorium]
MFHFLLLFVCFELIHCAPQRNVRLPEVNPSALPQKPSTPQQLQPQLNGQPRNSPQELFQLQPQAPNVRPQDQSAFPSEGPNENNRQHHNQPQQPPPPLPPIGPPQISWGQCPQLEPTMEEKIKKAAVITKCLEL
TPVPTNITRESVELHREQVAACALQMEGWFNEKGTYRFDKAESEIKNKKLQKDIENRVLTYHKQCKEEAEEKFPISSQNLIAQIQLYQACMDYFISEVCGIEVGPSAPAEF

>Dpte_XP_027195173 .1 GATA zinc finger domain-containing protein 10-like [Dermatophagoides pteronyssinus]
MFRSIIFTLLLAMMMFIIVDANIPNHYHHQQHNTSIKLASSMQQPSTQSIPLQASASQQSNTIMNKQQQQQHSHQQHQQPTLLTLEQQQDLLEQLAQLQRQTQQLLAAQQSLAAVAAAAAVNNNGDANSQTGG
NIPASGSANIGHLHHHQHQQQQPNHAQQFGRQQSINQSNTRSSSSSSSSSNVGHNNKPIQLQNTNGSDGNNNNSGNNNNNGNSQTTAINNGLPQSPQINWGKCPELEPSEQEKLAKANVITKCLETTPLPTNIT
RESVEQHREQIAACALRSEGWFTQQGGYDFSKAEKEIKNKRLQEDLENQVLNYHSQCRVESEDKYPTSTNSSIIAQIQLYQACMDYFISDVCGIEVNDSDVPQF
>Dpte_XP_027194279 .1 uncharacterized protein LOC113788996 [Dermatophagoides pteronyssinus]
MKFSTIFAITLATSSMMMMTVMAANKKNKIVAAASEPSTVSVQPADQTPRRPKINWGRCPQLEPTERDIESKSNILQECLRQNPPPQSESITQEQIIQHQQKVTECALKIENWFDKNGEYKYSKAEKEIKNKNL
AKDMEKKLLESHKQCSQIAKDRSQAVKNMTIVEQVQVYQGCMDAHITQNCQIEISAV

>Ssca_KPM10909 .1 hypothetical protein QR98_0094740 [Sarcoptes scabiei]
MIANMNLYALAIILLLAHLCDAQSTEKLDKSKLKQQQQLPQRPKIDWGKCPQLEPSEKDIKSKSNILQRCLKENPPPNQDSITPELIIGHQKNVTECALKIENWFDKKGDYKFEKAEKEIKNKKLSSEMQKALV
GSHQDCAEVSKTQKKQSKTPMDVVEQVQIYQGCMDAHITQHCQIKIGA
>SscaOBP_KPM10930 .1 hypothetical protein QR98_0094950 [Sarcoptes scabiei]
MMAIHQLFLTTVASLWSVMLMTAVVALPQPLNTSTNNKPADSISTASSSSIDSNVPLMASASSPSGQNLRSSSQQPMLLTLEQQQELLDQLAQLQRQTQQLLAAQQSLAAVAAAAAVNGNNNGVSIGTPLDGNS
NNDAGSHSPITSGSIQYHRQQNQQQTHPTTQQLNNPTSNQQSAAIRNDNIDQGSLEQSQSKSNQLNQMINNAQNSQSQSMSGLPQSPQINWGKCPELEPSEQEKLAKANVITKCLETTPLPANITRESVEQHRE
QIAGCALRSEGWFTPNGGYDFSKAEKEIRNKHLQADLENQVLNYHGQCRVESEEKYPTSNSSIIAQIQLYQACMDYFISDVCGIEVNDTDSPQF

>Rmay_OTF72123 -.1 hypothetical protein BLA29_010316, partial [Euroglyphus maynei]
QLPRAPKINWGRCPQLEPTQRDIEAKSSILQECLRQNPPPQSESITQEQIIQREYKYEKAEKEIKNKNLAKEMEKVLLESHKQCSQIAKDRKQAVKNLSNVEQVQIYQGCMDAHITQNCQIQISAV

**Supplementary Table S2. Assigned disulfide bridged-peptides identified in the tryptic/chymotryptic digest of OBP1, OBP2 and OBP5 from *Varroa destructor***. Reported are the protein under investigation, amino acid sequence, amino acid position within corresponding protein sequence, experimental/theoretical mass value of the observed parent ion, modified cysteine residue, and software used for characterization. § Cysteine involved in disulfide bridge; * bridged or linear peptide with aspecific cut; # software used for identification.

## *Varroa destructor* OBP1

| Assigned disulfide bridged-peptides | Position | $MH_2^{2+}$ (exp./theor.) | $MH_3^{3+}$ (exp./theor.) | $MH_4^{4+}$ (exp./theor.) | Cys-Cys | $MH^+$ (exp./theor.) | BioPharma Finder# | pLink# |
|---|---|---|---|---|---|---|---|---|
| [C§EQL]-[NISQIC§GIK] | (22-25)-(137-145) | 732.8628/ 732.8631 | 488.9112/ 488.9113 | | 22-142 | 1464.7179/ 1464.7184 | x | x |
| [C§EQLK]-[NISQIC§GIK] | (22-26)-(137-145) | 796.9125/ 796.9106 | 531.6109/ 531.6097 | 398.9601/ 398.9592 | 22-142 | 1592.8172/ 1592.8134 | x | x |
| [GKC§EQL]-[NISQIC§GIK] | (20-26)-(137-145) | 825.4242/ 825.4218 | | | 22-142 | 1649.8407/ 1649.8358 | x | |
| [C§EQLKPSESER]-[NISQIC§GIK] | (22-32)-(137-145) | 1139.5628/ 1139.5622 | 760.0444/ 760.0440 | 570.2853/ 570.2850 | 22-142 | 2278.1177/ 2278.1165 | x | x |
| [C§EQLKPSESER]-[IC§GIK]* | (22-32)-(141-145) | 918.4556/ 918.4534 | | | 22-142 | 1835.9035/ 1835.8989 | x | x |
| [C§EQLKPSESER]-[NISQIC§GI]* | (22-32)-(137-144) | 1075.5158/ 1075.5146 | 717.3465/ 717.3457 | | 22-142 | 2150.0238/ 2150.0215 | | x |
| [C§EQLKPSES]-[NISQIC§GIK]* | (22-30)-(137-145) | 996.9930/ 996.9903 | | | 22-142 | 1992.9782/ 1992.9728 | | x |
| [C§EQLKPSESER]-[DYNISQIC§GIK]* | (22-32)-(135-145) | 1278.6089/ 1278.6072 | 852.7419/ 852.7408 | | 22-142 | 2556.2100/ 2556.2067 | x | x |
| [C§EQLK]-[ISQIC§GIK]* | (22-26)-(138-145) | 739.8908/ 739.8892 | | | 22-142 | 1478.7738/ 1478.7705 | | x |
| [C§EQLKPS]-[DYNISQIC§GIK]* | (22-28)-(135-145) | 1028.0010/ 1027.9981 | | | 22-142 | 2054.9941/ 2054.9884 | x | x |
| [C§EQLKPSESER]-[ISQIC§GIK]* | (22-32)-(138-145) | 1082.5434/ 1082.5407 | 722.0315/ 722.0297 | | 22-142 | 2164.0790/ 2164.0736 | x | x |
| [C§EQLKPS]-[SQIC§GIK]* | (22-28)-(139-145) | 775.3918/ 775.3895 | | | 22-142 | 1549.7758/ 1549.7712 | | x |
| [C§EQLKPS]-[NISQIC§GIK]* | (22-28)-(137-145) | 888.9557/ 888.9530 | | | 22-142 | 1776.9036/ 1776.8982 | x | x |
| [C§EQLK]-[DYNISQIC§GIK]* | (22-26)-(135-145) | 935.9588/ 935.9558 | 624.3085/ 624.3064 | | 22-142 | 1870.9099/ 1870.9037 | x | x |
| [C§EQLKPSESER]-[SQIC§GIK]* | (22-32)-(139-145) | | 684.3367/ 684.3350 | | 22-142 | 2050.9944/ 2050.9895 | x | x |
| [C§L]-[ESVTTC§AL] | (42-43)-(64-71) | 528.2432/ 528.2413 | | | 42-69 | 1055.4787/ 1055.4748 | x | |
| [C§LQSLPLPDPEK]-[C§ALK]* | (42-53)-(69-72) | 885.9632/ 885.9603 | 590.9781/ 590.9761 | | 42-69 | 1770.9187/ 1770.9128 | x | x |
| [C§LQ]-[ESVTTC§AL]* | (42-44)-(64-71) | 592.2726/ 592.2708 | | | 42-69 | 1183.5375/ 1183.5338 | x | |
| [C§LQ]-[C§ALK]* | (42-44)-(69-72) | 397.6993/ 397.6988 | | | 42-69 | 794.3908/ 794.3899 | x | x |
| [HDAC§QK]-[QAC§MDY] | (109-114)-(131-136) | 714.7718/ 714.7709 | 476.8504/ 476.8499 | | 112-133 | 1428.5357/ 1428.5340 | x | x |
| [HDAC§QK]-[Q(pGlu)AC§MDY] | (109-114)-(131-136) | 706.2602/ 706.2576 | | | 112-133 | 1411.5125/ 1411.5075 | | x |
| [HDAC§QK]-[YQAC§MDY]* | (109-114)-(130-136) | 796.3044/ 796.3026 | | | 112-133 | 1591.6010/ 1591.5973 | | x |
| [HDAC§QK]-[AC§MDY]* | (109-114)-(132-136) | 650.7428/ 650.7416 | | | 112-133 | 1300.4778/ 1300.4754 | | x |

## *Varroa destructor* OBP2

| Assigned disulfide bridged-peptides | Position | $MH_2^{2+}$ (exp./theor.) | $MH_3^{3+}$ (exp./theor.) | $MH_4^{4+}$ (exp./theor.) | Cys-Cys | $MH^+$ (exp./theor.) | BioPharma Finder# | pLink# |
|---|---|---|---|---|---|---|---|---|
| [C§VQ]-[EDVTTC§ALK]* | (36-38)-(58-66) | 663.3096/ 663.3076 | 442.5423/ 442.5410 | | 36-63 | 1325.6113/ 1325.6075 | x | x |
| [C§VQ]-[REDVTTC§AL]* | (36-38)-(57-65) | 677.3122/ 677.3107 | | | 36-63 | 1353.6166/ 1353.6136 | | x |
| [C§VQ]-[HREDVTTC§AL]* | (36-38)-(56-65) | 745.8427/ 745.8402 | | | 36-63 | 1490.6776/ 1490.6726 | | x |
| [C§VQ]-[HREDVTTC§ALK]* | (36-38)-(56-66) | 809.8926/ 809.8876 | | | 36-63 | 1618.7774/ 1618.7675 | | x |
| [HAEC§QK]-[QAC§MDF] | (103-108)-(126-131) | 713.7839/ 713.7813 | 476.1919/ 476.1901 | | 106-128 | 1426.5600/ 1426.5548 | x | x |
| [HAEC§QK]-[Q(pGlu)AC§MDF] | (103-108)-(126-131) | 705.2706/ 705.2680 | | | 106-128 | 1409.5334/ 1409.5282 | | x |

## *Varroa destructor* OBP5

| Assigned disulfide bridged-peptides | Position | $MH_2^{2+}$ (exp./theor.) | $MH_3^{3+}$ (exp./theor.) | $MH_4^{4+}$ (exp./theor.) | Cys-Cys | $MH^+$ (exp./theor.) | BioPharma Finder# | pLink# |
|---|---|---|---|---|---|---|---|---|
| [TSNWGEC§EPLK]-[TC§PPNF] | (23-33)-(144-149) | 969.9248/ 969.9219 | 646.9524/ 646.9505 | | 29-145 | 1938.8417 /1938.8360 | x | x |
| [GEC§EPLK]-[TC§PPNF] | (27-33)-(144-149) | 725.8226/ 725.8209 | 484.2177/ 484.2165 | | 29-145 | 1450.6375/ 1450.6340 | x | x |
| [FTSNWGEC§EPLK]-[TC§PPNF] | (22-33)-(144-149) | 1043.4600/ 1043.4561 | 695.9759/ 695.9733 | | 29-145 | 2085.9121/ 2085.9044 | x | x |
| [TSNWGEC§EPL]-[TC§PPNF] | (23-32)-(144-149) | 905.8768/ 905.8744 | | | 29-145 | 1810.7457/ 1810.7410 | x | x |
| [FTSNWGEC§EPL]-[TC§PPNF] | (22-32)-(144-149) | 979.4106/ 979.4086 | | | 29-145 | 1957.8133/ 1957.8094 | x | x |
| [GEC§EPL]-[TC§PPNF] | (27-32)-(144-149) | 661.7748/ 661.7734 | | | 29-145 | 1322.5419/ 1322.5391 | x | x |
| [TSNWGEC§EPLK]-[TC§PPNFQ]* | (23-33)-(144-150) | 1033.9544/ 1033.9512 | | | 29-145 | 2066.9010/ 2066.8945 | | x |
| [NWGEC§EPLK]-[TC§PPNFQ]* | (25-33)-(144-150) | 939.9124/ 939.9113 | | | 29-145 | 1878.8171/ 1878.8148 | | x |
| [GEC§EPLK]-[TC§PPNFQ]* | (27-33)-(144-150) | 789.8514/ 789.8502 | | | 29-145 | 1578.6951/ 1578.6926 | x | x |
| [NWGEC§EPLK]-[TC§PPNF]* | (25-33)-(144-149) | 875.8844/ 875.8820 | | | 29-145 | 1750.7610/ 1750.7563 | | x |
| [C§R]-[C§MLK] | (50-51)-(71-74) | 385.1809/ 385.1759 | | | 50-71 | 769.3540/ 769.3518 | x | |
| [C§SQR]-[VEC§IF] | (114-117)-(134-138) | 550.7478/ 550.7470 | 367.5011/ 367.5006 | | 114-136 | 1100.4878/ 1100.4863 | x | x |
| [C§SQR]-[IYVEC§IF] | (114-117)-(132-138) | 688.8225/ 688.8208 | 459.5509/ 459.5498 | | 114-136 | 1376.6372/ 1376.6337 | x | x |
| [C§SQR]-[VEC§IFR] | (114-117)-(134-139) | 628.7996/ 628.7976 | | | 114-136 | 1256.5913/ 1256.5874 | x | x |
| [C§SQR]-[IYVEC§IFR] | (114-117)-(132-139) | | 511.5853/ 511.5834 | 383.9410/ 383.9395 | 114-136 | 1532.7404/ 1532.7347 | x | x |
| [C§SQR]-[EC§IF]* | (114-117)-(135-138) | 501.2140/ 501.2128 | | | 114-136 | 1001.4202/ 1001.4179 | x | x |
| [C§SQR]-[EC§IFR]* | (114-117)-(135-139) | 579.2652/ 579.2634 | | | 114-136 | 1157.5227/ 1157.5190 | x | x |
| [C§SQR]-[IYVEC§I]* | (114-117)-(132-137) | 615.2885/ 615.2865 | 410.5283/ 410.5269 | | 114-136 | 1229.5692/ 1229.5652 | | x |

**Supplementary Table S3**. Ligands tested with the four OBPs of *V. destructor*. Ligands are arranged in order of number of carbon atoms (No C). Tested ligands with each of the four proteins are marked by "v".

| No C | Ligand | OBP1 | OBP2 | OBP4 | OBP5 |
|---|---|---|---|---|---|
| | | Tryptophan quenching | | Competitive binding | |
| | *Terpenoids* | | | | |
| 8 | Vanillin | v | v | v | |
| 10 | Safranal | v | v | v | v |
| 10 | Coniferyl aldehyde | v | v | v | v |
| 10 | (+) Carvone | v | | | |
| 10 | (-) Carvone | v | | | |
| 10 | Citral | | | | v |
| 13 | β-Ionone | v | v | v | v |
| 13 | Geranyl acetone | | | v | v |
| 15 | Farnesol | v | | | v |
| 10 | Ocimene | | | v | |
| | | | | | |
| | *Aromatics* | | | | |
| 9 | Homovanillyl alcohol | v | v | v | v |
| 8 | Piperonyl alcohol | v | | | |
| 8 | Methyl p-hydroxybenzoate | v | v | v | v |
| 10 | Carvacrol | v | v | | |
| 7 | 4-Methylcathecol | v | v | | |
| 13 | Methoxybiphenyl | v | v | | |
| 7 | Benzyl cyanide | v | v | | |
| 10 | Eugenol | v | | v | v |
| 10 | Thymol | v | v | | |
| 9 | 4-Isopropylphenol | v | | | |
| 11 | Methyleugenol | v | v | | |
| 9 | Methoxyvinylphenol | v | v | | |
| 11 | *p-tert*-Butylbenzaldehyde | v | v | | v |
| 10 | Methoxycinnamaldehyde | v | v | | |
| 13 | Cyclamen aldehyde | v | v | | |
| 11 | Methoxyeugenol | v | | | |
| 14 | α-Pentylcinnamaldehyde | v | v | v | v |
| | | | | | |
| | *Acids, esters, aldehydes, alcohols* | | | | |
| 10 | Decanoic acid | | | | v |
| 10 | Decanal | | | | v |
| 10 | 9-Ketodecenoic acid | v | v | v | v |
| 12 | Dodecanoic acid | | | v | |
| 12 | 1-Dodecanol | | | v | |
| 12 | Ethyl decanoate | | | | v |
| 16 | *Z*11-Hexadecenal | | | v | v |
| 16 | Ethyl myristate | | | | v |
| 18 | Ethyl palmitate | | | | v |
| 18 | Oleic acid | | | v | v |

**Supplementary Table S4**. Primers used for cloning and expression of varroa OBPs. Restriction sites are underlined, stop codons are in bold.

| Name | Sequence |
|---|---|
| VdesOBP1-Nde | CGCATATGGCCCCTCAGGCACCTGC |
| VdesOBP1-Eco | CGGAATTC**CTA**AACCATGACTTTGATACC |
| VdesOBP2-Nde | CGCATATGGGCCCCGAGGCCGGAAA |
| VdesOBP2-Eco | CGGAATTC**TTA**AGATACAACCTTAATACC |
| VdesOBP3-Nde | CGCATATGGATACCGCCTCCGGAGA |
| VdesOBP3-Eco | CGGAATTC**TTA**CTTTGACGCCAACTTACA |
| VdesOBP4-Nde | CGCATATGGATACTGCTACAGGGGATCA |
| VdesOBP4-Eco | CGGAATTC**CTA**GCCTTGAGCCTTTACGGC |
| VdesOBP5-Nde | CGCATATGGGACCTCTGGCCAAGGA |
| VdesOBP5-Eco | CGGAATTC**TTA**CTGCGGGCCGCCGCTGCCATTG |

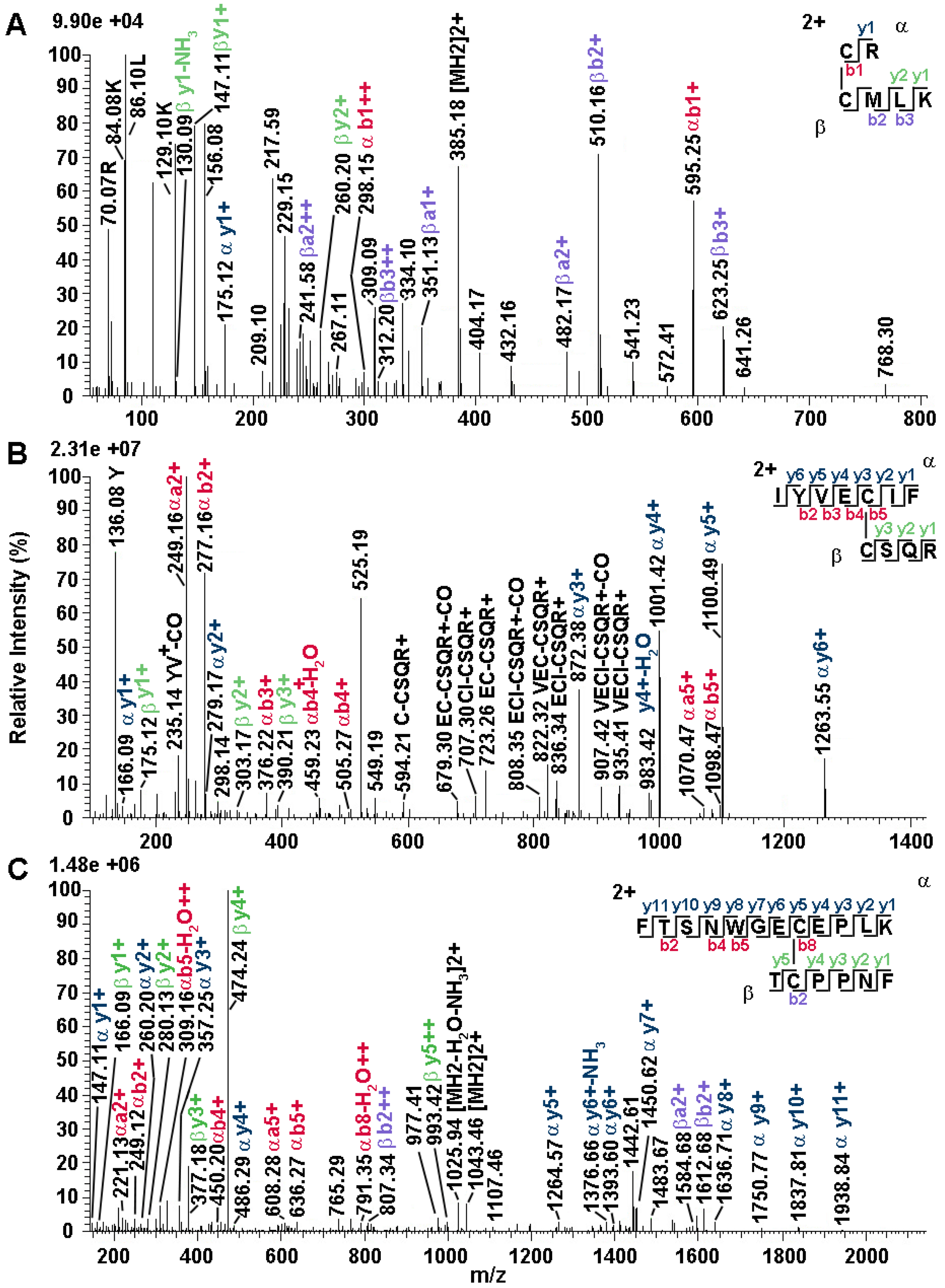


**Supplementary Figure S1. Fragmentation spectra of disulfide-bridged peptides identified in the tryptic-chymotryptic digest of *V. destructor* VdesOBP5 as revealed by nanoLC-ESI-Q-Orbitrap-MS/MS analysis.** The fragments are highlighted in different colours depending on peptides present in S-S-linked species, and on the corresponding b and y ion series. Complete data on disulphide-bridged peptides are reported in Supplementary Table S2 online.

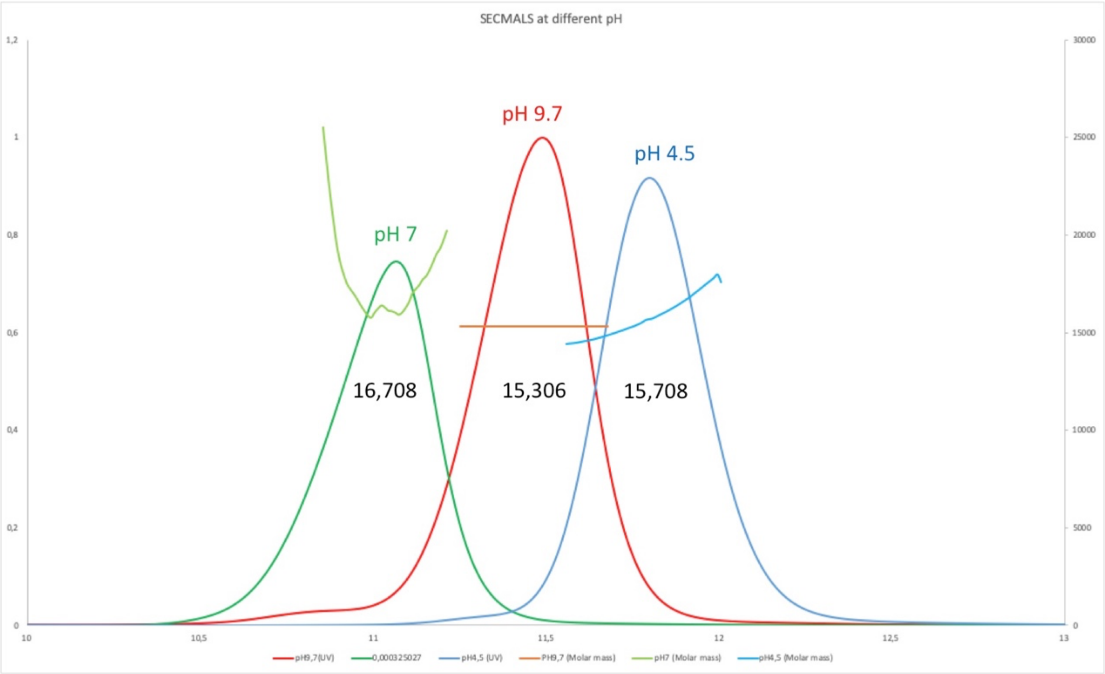


**Supplementary Fig. S2.** Analysis of VdesOBP1 aggregation in solution at different pH values, namely 4.5, 7.0 and 9.7, using HPLC with multi-angle laser light scattering /UV/ refractometry (SEC-MALLS-RI) coupled detectors (Wyatt, Santa-Barbara, USA). Mass values of 15,708 Da (pH 4.5, blue), 16,143 Da (pH 7.5, green) and 15,306 Da (pH 9.7, red) were measured.

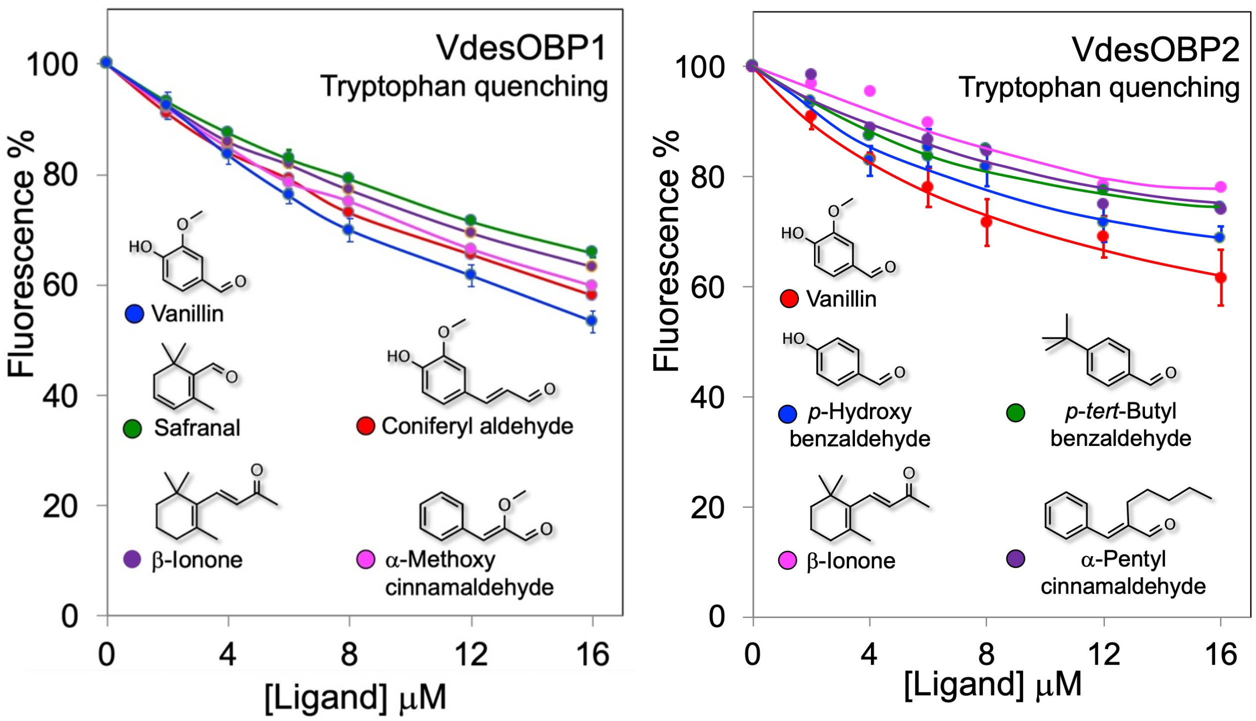


**Supplementary Fig. S3. Ligand-binding assays with VdesOBP1 and VdesOBP2**. With these two proteins we could not observe any blue shift in the fluorescence emission spectrum of 1-NPN in the presence of the protein. Therefore, we monitored the quenching of tryptophan intrinsic fluorescence caused by the entrance of a ligand in the binding pocket. Only chemicals capable of energy transfer were used in such assay. Tryptophan was excited at 295 nm and emitted fluorescence was measured around 335-340 nm.

**Supplementary Fig. S4**. Predicted phosphorylation sites (highlighted in blue) in the sequences of the five varroa OBPs. The six conserved cysteines are highlighted in yellow. The software used was NetPhos 3.1, which predicts serine, threonine and tyrosine potential phosphorylation sites in eukaryotic proteins. The predictions are relative to the following kinases: ATM, CKI, CKII, CaM-II, DNAPK, EGFR, GSK3, INSR, PKA, PKB, PKC, PKG, RSK, SRC, cdc2, cdk5 and p38MAPK.

Reference: Blom, N., Gammeltoft, S. & Brunak, S. Sequence- and structure-based prediction of eukaryotic protein phosphorylation sites. *J. Mol. Biol*. **294**, 1351-1362 (1999).

```
VdesOBP1 -------APQAPASATPAKVPVIEWGKCEQLK-PSESERTSKAAVVDKCLQSLPLPDPEK
VdesOBP2 -------------GPEAGKPPTIEWGKCSQLK-PSDSERMTKAAVVEKCVQSLPLPEAGK
VdesOBP3 --------DTASGDHASDTSQFGKWRTCLADK-LPAK----KREVFDECFI--------K
VdesOBP4 --------DTATGDHQPDSNQFGRWRTCLKSK-IPEN----KQPGMEACIN--------K
VdesOBP5 GPLAKDKSERITPKQTVEEFFTSNWGECEPLKNPLVLPEISDAATWERCRVHIP------

VdesOBP1 ATQQEIDKHRESVTTCALKAEGWFDDEGVYKFDRARNEIKNKKLDSEVEEAVLLKHDACQ
VdesOBP2 ASQAEIEKHREDVTTCALKAEGWFDDKGVYKFDRARDEIKNKKLASDIETPVLEKHAECQ
VdesOBP3 PGGTDMNKFRRGLN-CVLTSD------GLLKNHKVDLKKMSMTAASAGSPELKKAYENCP
VdesOBP4 PGGTDMSKFRRGLG-CVLESY------GIVNNHRVDLTKMRSSASLVTSPELKKAFMECP
VdesOBP5 LSPDQVEEALPNLSRCMLKVIGWVRANGNMNLTKYMDYLSQAGLDDKTLELTKTAHEKCS

VdesOBP1 KEATEKHD-DYINQVQLYQACMDYNISQICGIKVMV------------------------
VdesOBP2 KEAGEKHTSDYIKQVQLYQACMDFNISQICGIKVVS------------------------
VdesOBP3 KD-------DKNTSEDRSIKCLIDHLEVDCKLASK-------------------------
VdesOBP4 KD-------DKNSSLDRSIKCVIDHLETSCSAVKAQG-----------------------
VdesOBP5 QRALSDGSGDYKEQDKIYVECIFRHFDKTCPPNFQAAVKQGYIVGITQISFVPRYDEVEV

VdesOBP5 ESDTDAETEGQAGPSVADATGEAAANDNANGSGGPQ
```
